# A systematic review and meta-analysis of geographic differences in comorbidities and associated severity and mortality among individuals with COVID-19

**DOI:** 10.1038/s41598-021-88130-w

**Published:** 2021-04-20

**Authors:** Bhaskar Thakur, Pallavi Dubey, Joseph Benitez, Joshua P. Torres, Sireesha Reddy, Navkiran Shokar, Koko Aung, Debabrata Mukherjee, Alok Kumar Dwivedi

**Affiliations:** 1 Division of Biostatistics and Epidemiology, Department of Molecular and Translational Medicine, Paul L. Foster School of Medicine, Texas Tech University Health Sciences Center El Paso, 5001 El Paso Drive, El Paso, TX 79905 USA; 2 Department of Obstetrics and Gynecology, Paul L. Foster School of Medicine, Texas Tech University Health Sciences Center El Paso, 4800 Alberta Avenue, El Paso, TX 79905 USA; 3 Graduate School of Biomedical Sciences, Texas Tech University Health Sciences Center El Paso, 5001 El Paso Drive, El Paso, TX 79905 USA; 4 Department of Family and Community Medicine, Paul L. Foster School of Medicine, Texas Tech University Health Sciences Center El Paso, 9849 Kenworthy St., El Paso, TX 79924 USA; 5 Department of Internal Medicine, Paul L. Foster School of Medicine, Texas Tech University Health Science Center El Paso, 4800 Alberta Ave, El Paso, TX 79905 USA; 6 Biostatistics and Epidemiological Consulting Lab, Texas Tech University Health Sciences Center El Paso, 5001 El Paso Drive, El Paso, Texas 79905 USA

**Keywords:** Cardiology, Health care, Medical research, Risk factors

## Abstract

Several comorbidities have been shown to be associated with coronavirus disease 2019 (COVID-19) related severity and mortality. However, considerable variation in the prevalence estimates of comorbidities and their effects on COVID-19 morbidity and mortality have been observed in prior studies. This systematic review and meta-analysis aimed to determine geographical, age, and gender related differences in the prevalence of comorbidities and associated severity and mortality rates among COVID-19 patients. We conducted a search using PubMed, Scopus, and EMBASE to include all COVID-19 studies published between January 1st, 2020 to July 24th, 2020 reporting comorbidities with severity or mortality. We included studies reporting the confirmed diagnosis of COVID-19 on human patients that also provided information on comorbidities or disease outcomes. We used DerSimonian and Laird random effects method for calculating estimates. Of 120 studies with 125,446 patients, the most prevalent comorbidity was hypertension (32%), obesity (25%), diabetes (18%), and cardiovascular disease (16%) while chronic kidney or other renal diseases (51%, 44%), cerebrovascular accident (43%, 44%), and cardiovascular disease (44%, 40%) patients had more COVID-19 severity and mortality respectively. Considerable variation in the prevalence of comorbidities and associated disease severity and mortality in different geographic regions was observed. The highest mortality was observed in studies with Latin American and European patients with any medical condition, mostly older adults (≥ 65 years), and predominantly male patients. Although the US studies observed the highest prevalence of comorbidities in COVID-19 patients, the severity of COVID-19 among each comorbid condition was highest in Asian studies whereas the mortality was highest in the European and Latin American countries. Risk stratification and effective control strategies for the COVID-19 should be done according to comorbidities, age, and gender differences specific to geographical location.

## Introduction

The world health organization (WHO) officially declared the novel coronavirus (COVID-19) infected by SARS-CoV-2 (severe acute respiratory syndrome coronavirus 2) as a global pandemic on March 11th 2020 because of the alarming level of transmission, the evolving disparities among cases, and rising mortality rates^1^. The current strategies for managing COVID-19 are mainly with treatments that showed promising results in observational or preliminary clinical trial studies or recommended for an earlier outbreak of SARS-CoV-1. Given the nature of transmission of SARS-CoV-2 and the lack of any available antiviral therapy or vaccine, the optimal approach to treatment remains unclear. Rapidly evolving data provides guidance on therapeutic approach, including risk stratification utilizing comorbidities and laboratory features associated with severe COVID-19, prevention of venous thromboembolism, empiric treatment for bacterial pneumonia, addressing hypoxia and use of immunomodulatory agents on a case-by-case basis^2–5^.

Numerous studies have identified comorbidities associated with the adverse prognosis of COVID-19. Age, gender, and at least a few comorbidities have been shown to be the strongest predictors of prognosis of COVID-19 patients^6–10^. Prior systematic reviews and meta-analyses confirmed that diabetes mellitus (DM) and hypertension (HTN) are strongly associated with mortality in COVID-19 patients^11^ and most frequently observed conditions in COVID-19^12^. However, malignancy and DM were found to be associated with disease severity in prior meta-analyses^13,14^. Other meta-analyses^11,14,15^ showed that chronic kidney disease (CKD), chronic obstructive pulmonary disease (COPD), cardiovascular disease (CVD), DM, and HTN are the major risk factors predicting severity in COVID-19 patients. In contrast, a large study identified CKD as the major risk factor for mortality while COPD as the risk factor for the severity of COVID-19^9^. Another meta-analysis^16^ showed that cerebrovascular disease (CVA) is strongly associated with the severity and mortality in COVID-19. Most of these reviews were based on the analysis of studies where the majority of participants were from China. Recently, several observational studies from the US, Europe, and Latin America also demonstrated some coexisting diseases associated with the severity and mortality among COVID-19 patients^17–19^. Some studies^20–22^ reported COPD, CKD, malignancy, HTN, and DM as the prominent risk factors for disease severity or mortality in COVID-19 patients while other studies^23–29^ showed no association between these comorbidities and the disease outcomes. These studies at times reported rather inconsistent and conflicting findings. Rapid accumulation of data on comorbidities associated with poor prognosis of COVID-19 warrant updating the evidence.

The continental differences in the distribution of COVID-19 comorbidities, disease severity and mortality are more likely due to the variation in the timing of COVID-19 spread, population size, and prevalence of comorbidities among COVID-19 patients, age and gender distribution, implementation of mitigation strategies, diagnostic testing, capacity, COVID-19 management strategies, and reporting accuracy^30,31^. A proper understanding of coexisting diseases associated with COVID-19 severity and mortality is paramount to effectively allocate healthcare resources, endorse appropriate preventive and containment measures, and guide emerging treatment protocols. We sought to perform a comprehensive systematic review and meta-analysis to determine the differences in the prevalence of major comorbidities associated with COVID-19 and the severity and mortality of COVID-19 associated with each of the comorbidities according to the geographical regions. In addition, we also aimed to determine the impact of age and gender on the distribution of different comorbidities, severity, and mortality among COVID-19 patients.

## Methods

### Data sources and search strategy

We followed the preferred reporting in systematic reviews and meta-analyses (PRISMA)^32^ guidelines and SAMBR^33^ checklists for conducting and reporting this study. We conducted a systematic literature search using multiple electronic databases including PubMed, Scopus, and EMBASE between Jan 1st, 2020 to July 24th, 2020. The search strategy was designed to retrieve all published articles on COVID-19 reporting comorbidities and outcomes in the reported comorbidities. We applied various combinations of Boolean operators by using the following keywords for our search: [(SARS-Cov-2 OR 2019-nCOv OR COVID-19 OR coronavirus) AND (comorbidities OR comorbid OR comorbidity) AND (“severity” OR “secondary infection” OR “critical care” OR “ICU” OR intensive care unit” OR “fatality rate” OR “Death” OR “Mortality”)]. In addition, the references from review or other systematic review studies were cross-checked to retrieve any additional articles that were missed at the initial search. Three persons (BT, JT, and JB) reviewed all articles to eliminate any duplicated study. Studies with similar authors, the duration of data collection, and the location of the study were strictly matched to further identify any duplicated study. All the duplicates were omitted from the analyses.

### Study selection

Studies were eligible for inclusion in our systematic review if they met the following criteria: (1) originally published in the English language; (2) included confirmed diagnosis of COVID-19 through laboratory examination; (3) conducted on human patients; (4) provided information about comorbidities; (5) contained information on the disease outcomes: severity or mortality within comorbidity; and (6) published as an original investigation. Studies without diagnostic information, involved the pediatric population, and performed surgical procedures were excluded from the analysis. In addition, any studies published as case reports and case series with smaller numbers (≤ 10) were also excluded from the analyses.

### Data extraction

All relevant information from the eligible studies were extracted and recorded in an excel datasheet. The following information was extracted from each study: study characteristics involved the first author of the study; geographical region (US, Europe, Latin America, Asia, South America); sample size (total number of patients with COVID-19); the average age in years with standard deviation or interquartile range; gender (male and female ratio); status of the comorbidities (number of subjects without any comorbidity, number of subjects with one comorbidity, number of subjects with multiple comorbidities), the type of comorbidity that included HTN, DM, CVD, obesity, CVA, lung disease, cancer/malignancy, either chronic or acute kidney or other renal diseases (CKD) and liver disease, and the outcomes of COVID-19 (number of severe cases and number of fatalities) within each comorbid condition. The detail information on the inclusion of comorbidities, outcomes including the criteria for COVID-19 severity assessment, and comparing variables is provided in an additional (supplementary file).

### Quality assessment

Methodological Index for Non-Randomized Studies (MINORS) scale was used for critical appraisal of study quality^34^. Three authors (BT, JT, and JB) independently graded the quality of the included studies using Sundemo et al. 2019 methods^35^. Consensus discussions were carried out among the three authors to resolve any disagreements. Additional information on quality assessment and publication bias is included in the additional file (supplementary file).

### Statistical analysis

We estimated the pooled proportion of underlying medical conditions, severity, and mortality among each comorbidity separately by geographic region (Asia, Europe, Latin America, USA) using a random effects meta-analysis of proportions with DerSimonian and Laird (D&L) method^36^. A priori random effects model was applied due to considerable heterogeneity across the studies. We applied the Freeman-Tukey double arcsine method for computing a 95% confidence interval (CI) for each proportion to obtain a reasonable range. The pooled proportion of each comorbidity, severity, and mortality was estimated according to average age groups (≤ 50 years, 51 – 64 years, and ≥ 65 years) and percentage of the female population (≤ 50%: male dominant vs. > 50%: female dominant) using random effects meta-analysis of proportions with the D&L method. Meta-regression analyses with Knapp-Hartung modification were also performed to determine associations of demographic data with disease outcomes. All the estimated proportions were summarized with their 95%CI and I^2^ statistic^37^. An I^2^ greater than 50% was considered as an indication of presence of heterogeneity. All statistical analyses were conducted using Stata 15.1.

## Results

### Characteristics of included studies

**Figure 1:**
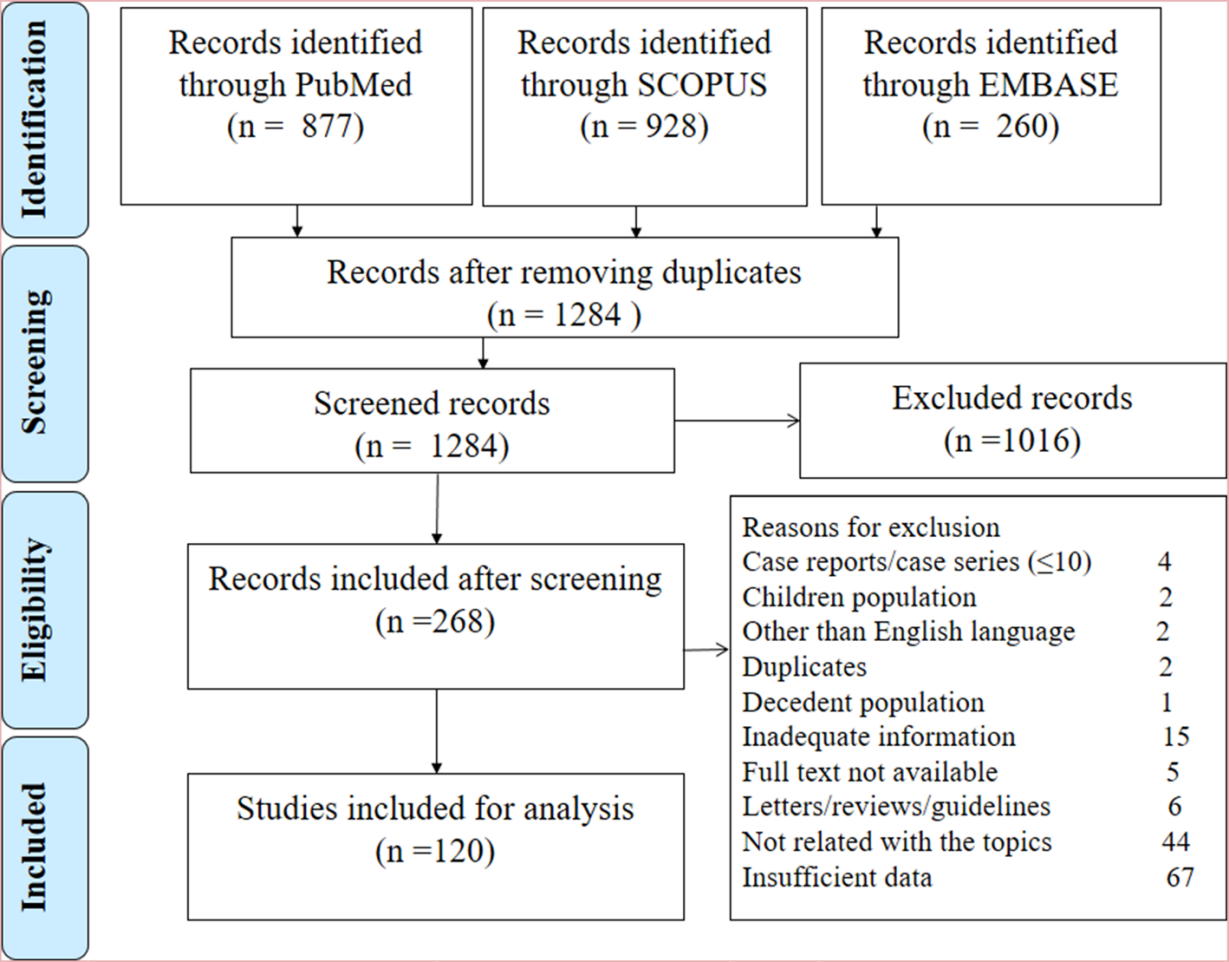
PRISMA flowchart for the selection of studies at different stages.

Figure 1 shows the search pattern and exclusion of articles with specific reasons at each step. Out of 120 eligible studies, 118 studies were conducted in 19 countries (China: 59; USA: 16; Italy: nine; Spain: five; Iran: five; India: three; South Korea: three; Brazil: three; Mexico: two; Switzerland: two; Turkey: two; UK: two; Denmark: one; France: one; Germany: one; Netherlands: one; Oman: one; Poland: one; and South Africa: one) and two studies represented multiple countries (one study comprised two European countries and another study comprised 13 Asian countries). In our quality appraisal, we observed a high quality of conducting and reporting of 101 studies (94.2%) and fair quality in the rest of the 19 studies (5.83%). The detail of study characteristics and quality assessment of each study is included in the additional file (Supplementary Tables 1 and 2). No evidence of publication bias or small sample size effect was observed for the estimation of prevalence of comorbidity or associated mortality except for the severity in some comorbidities (additional file: Supplementary Table 3).

Of the total, 72 studies reported from Asia, 26 from Europe, 16 from the USA, five from Latin America, and one from South Africa. These studies yielded a total sample size of 125,546 patients (Asia: 50.6%, Europe: 11.1%; USA: 16.4%; Latin America: 19.6%, and South Africa: 2.4%). The female proportion was estimated as 45% (95% CI: 43%, 47%) with the highest in South Africa followed by Asia, USA, and Latin America and the lowest in Europe. The mean age of the patients was 56.2 years (95% CI: 52.6 – 59.8 years) with the highest in Europe (64.8 years), followed by the USA (60.4 years), Asia (54.4 years), and Latin America (50.2 years) (additional file: Supplementary Table 4).

### Proportions of comorbid diseases

**Figure 2:**
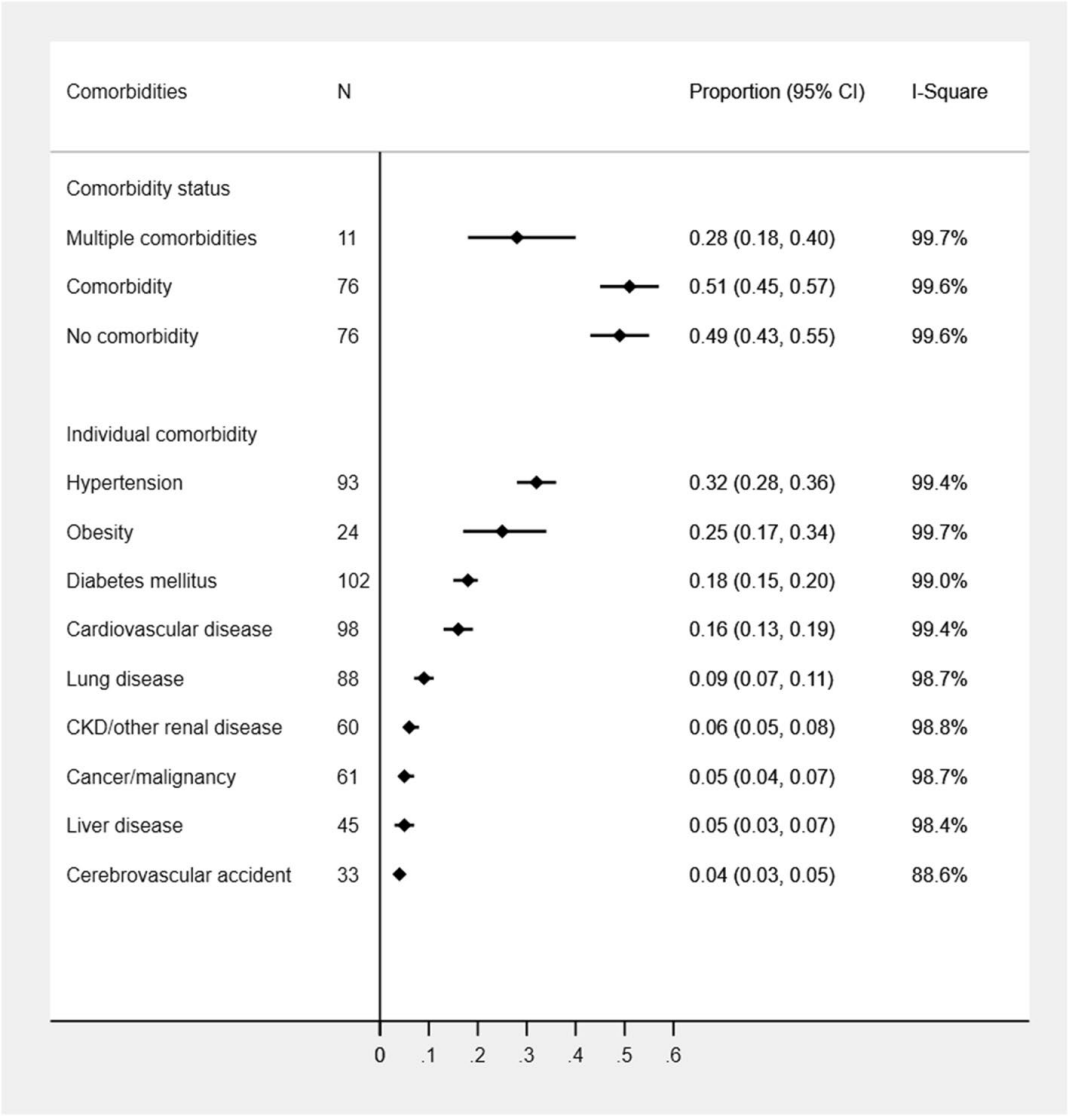
Prevalence of comorbidities among COVID-19 patients.

In the pooled estimation of all studies, over half of the COVID-19 cases had at least one comorbidity while less than one-third of COVID cases had multiple comorbidities (28%; 95%CI: 18–40%). The history of liver disease and CVA were frequently reported in Asian studies while rarely reported in other country studies. In contrast, obesity was rarely reported from Asian studies. The most prevalent comorbidity was HTN (32%; 95%CI: 95% CI: 28%, 36%) followed by obesity (25%; 95% CI: 17%, 34%), DM (18%; 95% CI: 15%, 20%), CVD (16%; 95% CI: 13%, 19%), lung disease (9%; 95%CI: 7–11%), and CKD (6%; 95%CI: 5–8%) while other underlying medical conditions were estimated to be 4–5% (Fig. 2). All the considered comorbidities except for the liver disease were frequently observed in the US studies (range: 10–51%) followed by South African (range: 9–42%), European (range: 2–40%), Latin American (range: 3–32%), and Asian (range: 3–25%) studies. HTN, DM, and CVD remained the top three medical conditions among COVID-19 cases in the USA (51%, 31%, and 32% respectively), Europe (40%, 20%, and 25% respectively), Latin America (32%, 18%, and 20% respectively) and Asia (25%, 14%, and 10% respectively) regions. The prevalence of multiple comorbidities was also observed highest in the US (48%) and European (40%) studies compared to Asian (23%) and Latin American (19%) studies. Significant differences in all comorbidities other than CVA and liver disease were observed between geographic regions (Table 1, additional file: Supplementary Table 5). All the estimates yielded a substantial presence of heterogeneity across studies (Table 1). Studies with older adults (≥ 65 years) were associated with the increased prevalence of most comorbidities (Table 2, additional file: Supplementary Table 6). However, no substantial difference in the proportion of any comorbidities between male and female-dominant studies was observed (Table 3).

**Table 1:** The proportion estimates of underlying medical diseases and associated COVID-19 severity and mortality by geographic region.

	Asia			Europe			Latin America			USA			*p* value
	N	P (95% CI)	I^2^	N	P (95% CI)	I^2^	N	P (95% CI)	I^2^	N	P (95% CI)	I^2^	
**Comorbidities**													
One comorbidity	56	0.43 (0.39, 0.47)	98.2	12	0.66 (0.60, 0.72)	95.7	2	0.45 (0.45, 0.46)	ID	6	0.87 (0.77, 0.95)	99.5	< 0.001
Multiple comorbidities	7	0.23 (0.14, 0.33)	97.6	1	0.40 (0.36, 0.44)	ID	1	0.19 (0.19, 0.20)	ID	2	0.48 (0.48, 0.49)	ID	0.183
HTN	58	0.25 (0.22, 0.28)	98.0	21	0.40 (0.33, 0.47)	97.8	1	0.32 (0.25, 0.41)	ID	12	0.51 (0.40, 0.61)	99.5	< 0.001
DM	62	0.14 (0.12, 0.16)	96.5	22	0.20 (0.17, 0.23)	92.7	4	0.18 (0.08, 0.33)	99.5	13	0.31 (0.26, 0.36)	98.1	< 0.001
CVD	56	0.10 (0.07, 0.13)	99.3	21	0.25 (0.20, 0.30)	96.7	4	0.20 (0.06, 0.40)	99.7	12	0.32 (0.26, 0.39)	98.8	< 0.001
Obesity	1	0.04 (0.03, 0.04)	ID	8	0.19 (0.14, 0.31)	94.9	4	0.08 (0.03, 0.17)	99.2	11	0.39 (0.33, 0.46)	98.3	0.002
CVA	29	0.04 (0.03, 0.05)	85.2	1	0.02 (0.00, 0.08)	ID	NA	NA	NA	3	0.10 (0.05, 0.15)	ID	0.283
Lung disease	52	0.06 (0.04, 0.08)	98.6	19	0.13 (0.09, 0.18)	97.6	4	0.04 (0.00, 0.12)	99.3	12	0.19 (0.15, 0.23)	97.0	< 0.001
Cancer/malignancy	41	0.03 (0.02, 0.04)	95.4	13	0.11 (0.08, 0.14)	93.0	NA	NA	NA	7	0.10 (0.08, 0.13)	95.1	< 0.001
CKD/other renal disease	33	0.03 (0.02, 0.03)	89.9	11	0.09 (0.03, 0.17)	99.2	3	0.03 (0.01, 0.07)	ID	12	0.19 (0.15, 0.23)	97.7	< 0.001
Liver disease	37	0.05 (0.03, 0.09)	98.4	2	0.02 (0.01, 0.02)	ID	2	0.01 (0.01, 0.01)	ID	3	0.03 (0.02, 0.04)	66.7	0.879
**Severity**													
Total	29	0.29 (0.24, 0.34)	96.0	4	0.30 (0.29, 0.30)	74.8	1	0.30 (0.29, 0.30)	ID	4	0.33 (0.18, 0.50)	99.5	0.176
One comorbidity	29	0.46 (0.39, 0.53)	92.4	4	0.21 (0.16, 0.27)	62.6	1	0.41 (0.40, 0.42)	ID	4	0.35 (0.22, 0.49)	99.0	0.119
No comorbidity	29	0.17 (0.13, 0.21)	91.0	4	0.33 (0.03, 0.75)	97.4	1	0.20 (0.19, 0.21)	ID	4	0.25 (0.08, 0.46)	98.0	0.604
Multiple comorbidities	5	0.56 (0.40, 0.71)	84.8	1	0.16 (0.11, 0.21)	ID	1	0.48 (0.46, 0.50)	ID	1	0.21 (0.19, 0.23)	ID	0.006
HTN	39	0.45 (0.40, 0.51)	88.8	12	0.29 (0.21, 0.37)	81.2	1	0.22 (0.11, 0.37)	ID	7	0.34 (0.24, 0.44)	97.3	0.044
DM	41	0.50 (0.44, 0.57)	85.9	11	0.31 (0.24, 0.38)	55.3	1	0.38 (0.21, 0.56)	ID	8	0.31 (0.23, 0.40)	94.5	0.057
CVD	39	0.55 (0.46, 0.63)	86.7	11	0.35 (0.20, 0.51)	92.8	1	0.17 (0.00, 0.64)	ID	7	0.31 (0.21, 0.43)	96.1	0.139
Obesity	1	0.24 (0.19, 0.29)	ID	6	0.37 (0.28, 0.47)	68.2	1	0.30 (0.18, 0.45)	ID	8	0.31 (0.21, 0.42)	97.1	0.877
CVA	15	0.47 (0.38, 0.56)	49.0	1	0.00 (0.00, 0.84)	ID	NA	NA	NA	2	0.23 (0.19, 0.29)	ID	0.069
Lung disease	32	0.52 (0.40, 0.65)	85.2	11	0.35 (0.27, 0.44)	42.0	1	0.25 (0.01, 0.81)	ID	8	0.29 (0.20, 0.40)	93.5	0.189
Cancer/malignancy	23	0.40 (0.29, 0.51)	53.5	7	0.29 (0.14, 0.46)	61.5	NA	NA	NA	4	0.38 (0.18, 0.60)	96.1	0.811
CKD/other renal disease	21	0.68 (0.46, 0.87)	86.3	6	0.36 (0.20, 0.54)	50.3	NA	NA	NA	7	0.33 (0.24, 0.44)	91.6	0.001
Liver disease	26	0.22 (0.14, 0.31)	54.3	1	0.50 (0.01, 0.99)	ID	1	0.50 (0.01, 0.99)	ID	1	0.35 (0.20, 0.54)	ID	0.815
**Mortality**													
Total	24	14 (0.10, 0.19)	98.6	5	0.31 (0.16, 0.48)	99.3	2	0.11 (0.11, 012)	ID	3	0.23 (0.11, 0.37)	ID	0.206
One comorbidity	24	0.24 (0.17, 0.31)	97.1	5	0.39 (0.23, 0.56)	98.9	2	0.18 (0.17, 0.19)	ID	3	0.26 (0.16, 0.36)	ID	0.391
No comorbidity	24	0.06 (0.03, 0.09)	97.4	5	0.14 (0.01, 0.38)	99.1	2	0.06 (0.05, 0.06)	ID	3	0.06 (0.01, 0.13)	ID	0.306
Multiple comorbidities	4	0.14 (0.03, 0.32)	95.2	NA	NA	NA	1	0.25 (0.23, 0.26)	ID	2	0.28 (0.27, 0.29)	ID	0.130
HTN	27	0.28 (0.21, 0.36)	96.7	10	0.36 (0.27, 0.46)	96.8	NA	NA	NA	7	0.28 (0.23, 0.34)	95.2	0.277
DM	30	0.27 (0.21, 0.34)	92.8	11	0.41 (0.32, 0.51)	92.2	3	0.55 (0.45, 0.64)	ID	7	0.28 (0.23, 0.33)	92.1	< 0.001
CVD	29	0.36 (0.28, 0.45)	91.3	12	0.44 (0.36, 0.52)	90.2	3	0.55 (0.47, 0.62)	ID	7	0.40 (0.31, 0.51)	97.1	0.035
Obesity	NA	NA	NA	3	0.37 (0.22, 0.53)	ID	3	0.51 (0.41, 0.61)	ID	6	0.17 (0.11, 0.24)	95.2	0.008
CVA	13	0.48 (0.29, 0.67)	80.2	NA	NA	NA	NA	NA	NA	2	0.39 (0.34, 0.45)	ID	0.672
Lung disease	24	0.33 (0.21, 0.47)	90.0	8	0.40 (0.28, 0.54)	89.5	3	0.53 (0.49, 0.57)	ID	6	0.24 (0.19, 0.30)	75.5	0.080
Cancer/malignancy	20	0.27 (0.13, 0.44)	75.1	6	0.44 (0.30, 0.58)	91.4	NA	NA	NA	4	0.30 (0.23, 0.37)	87.6	0.083
CKD/other renal disease	16	0.38 (0.20, 0.57)	85.0	5	0.60 (0.41, 0.79)	94.5	3	0.81 (0.61, 0.95)	ID	8	0.33 (0.27, 0.39)	84.9	0.001
Liver disease	14	0.27 (0.10, 0.47)	85.2	1	0.49 (0.38, 0.60)	ID	1	0.65 (0.54, 0.75)	ID	3	0.23 (0.17, 0.30)	ID	0.123

*N* Number of studies, *P* Proportion, *CI* Confidence interval, *ID* Insufficient data, *NA* Not available, *HTN* Hypertension, *DM* Diabetes mellitus, *CVD* Cardiovascular disease, *CVA* Cerebrovascular accident, *CKD* Chronic kidney disease, *I*^2^ Index for between studies heterogeneity.

**Table 2:** The proportion estimates of underlying medical diseases and associated COVID-19 severity and mortality by age groups.

	Age ≤ 50 years			50 years < Age ≤ 65 years			Age > 65 years			*p* value
	N	P (95% CI)	I^2^	N	P (95% CI)	I^2^	N	P (95% CI)	I^2^	
**Comorbidities**										
One comorbidity	20	0.34 (0.29, 0.40)	96.4	44	0.53 (0.45, 0.61)	99.4	10	0.75 (0.63, 0.85)	98.0	< 0.001
Multiple comorbidities	4	0.13 (0.07, 0.20)	98.5	5	0.43 (0.35, 0.50)	96.6	2	0.33 (0.29, 0.37)	ID	0.002
HTN	20	0.17 (0.13, 0.20)	94.4	52	0.33 (0.28, 0.38)	99.0	18	0.48 (0.39, 0.56)	98.0	< 0.001
DM	22	0.12 (0.09, 0.15)	93.8	56	0.18 (0.15, 0.21)	98.3	20	0.25 (0.19, 0.31)	97.3	< 0.001
CVD	21	0.10 (0.03, 0.20)	99.5	54	0.14 (0.11, 0.17)	98.8	19	0.30 (0.24, 0.37)	97.3	< 0.001
Obesity	5	0.13 (0.04, 0.27)	99.5	11	0.31 (0.18, 0.46)	99.7	7	0.27 (0.20, 0.35)	94.4	0.237
CVA	5	0.03 (0.01, 0.07)	95.4	23	0.04 (0.03, 0.05)	78.6	5	0.09 (0.05, 0.15)	88.0	0.170
Lung disease	20	0.05 (0.02, 0.10)	99.1	48	0.09 (0.07, 0.11)	97.9	16	0.14 (0.11, 0.18)	87.3	0.058
Cancer/malignancy	11	0.02 (0.01, 0.04)	89.5	37	0.05 (0.04, 0.07)	96.7	11	0.09 (0.06, 0.13)	92.0	0.013
CKD/other renal disease	11	0.03 (0.01, 0.05)	94.3	34	0.05 (0.03, 0.07)	98.3	12	0.17 (0.10, 0.24)	98.0	< 0.001
Liver disease	15	0.04 (0.01, 0.06)	96.0	24	0.06 (0.03, 0.09)	98.8	6	0.03 (0.01, 0.06)	80.8	0.703
**Severity**										
One comorbidity	16	0.39 (0.33, 0.45)	86.4	19	0.44 (0.36, 0.51)	97.3	3	0.37 (0.25, 0.50)	ID	0.961
No comorbidity	16	0.12 (0.08, 0.16)	94.4	19	0.26 (0.19, 0.34)	96.1	3	0.25 (0.09, 0.45)	ID	0.283
Multiple comorbidities	4	0.44 (0.39, 0.49)	57.1	4	0.46 (0.26, 0.67)	94.7	NA	NA	NA	0.001
HTN	18	0.40 (0.33, 0.47)	77.7	34	0.40 (0.35, 0.46)	93.8	7	0.36 (0.23, 0.50)	89.5	0.870
DM	19	0.46 (0.38, 0.54)	77.8	35	0.42 (0.36, 0.48)	90.8	7	0.35 (0.25, 0.46)	69.6	0.798
CVD	18	0.41 (0.31, 0.51)	77.8	33	0.46 (0.39, 0.54)	88.4	7	0.37 (0.20, 0.57)	93.5	0.713
Obesity	4	0.32 (0.19, 0.46)	93.3	8	0.31 (0.21, 0.41)	96.6	4	0.40 (0.28, 0.52)	66.3	0.742
CVA	5	0.50 (0.30, 0.69)	59.0	15	0.42 (0.30, 0.55)	71.1	1	0.45 (0.23, 0.68)		0.699
Lung disease	17	0.33 (0.23, 0.45)	71.6	28	0.46 (0.37, 0.55)	88.9	7	0.38 (0.28, 0.49)	25.5	0.653
Cancer/malignancy	11	0.47 (0.29, 0.65)	55.0	18	0.31 (0.21, 0.42)	81.4	4	0.50 (0.14, 0.86)	82.9	0.533
CKD/other renal disease	9	0.64 (0.26, 0.95)	94.5	21	0.47 (0.35, 0.60)	87.4	4	0.39 (0.29, 0.49)	30.1	0.239
Liver disease	14	0.19 (0.09, 0.31)	37.8	13	0.29 (0.14, 0.44)	66.1	2	0.37 (0.09, 0.70)	ID	0.883
**Mortality**										
One comorbidity	6	0.14 (0.06, 0.25)	97.3	21	0.27 (0.20, 0.35)	98.2	5	0.44 (0.31, 0.57)	93.9	0.035
No comorbidity	6	0.02 (0.00, 0.04)	95.9	21	0.08 (0.04, 0.13)	97.8	5	0.14 (0.03, 0.30)	93.4	0.113
Multiple comorbidities	4	0.14 (0.03, 0.31)	97.2	2	0.28 (0.27, 0.29)	ID	1	0.26 (0.18, 0.36)	ID	0.397
HTN	4	0.28 (0.10, 0.51)	96.0	27	0.31 (0.25, 0.38)	96.8	11	0.35 (0.28, 0.41)	91.1	0.730
DM	5	0.35 (0.15, 0.57)	95.0	30	0.31 (0.24, 0.39)	97.2	13	0.36 (0.31, 0.42)	74.7	0.772
CVD	6	0.34 (0.14, 0.56)	90.6	29	0.40 (0.33, 0.48)	95.8	13	0.47 (0.39, 0.54)	90.1	0.411
Obesity	1	0.50 (0.07, 0.93)	ID	6	0.21 (0.10, 0.34)	97.7	4	0.32 (0.23, 0.43)	80.2	0.663
CVA	1	0.20 (0.08, 0.39)	ID	10	0.50 (0.29, 0.71)	83.5	4	0.48 (0.38, 0.57)	0.0	0.363
Lung disease	5	0.36 (0.22, 0.5)	0.0	25	0.30 (0.22, 0.38)	92.5	9	0.45 (0.31, 0.59)	85.1	0.410
Cancer/malignancy	2	0.14 (0.03, 0.32)	ID	19	0.33 (0.22, 0.44)	88.1	7	0.40 (0.30, 0.50)	39.2	0.513
CKD/other renal disease	4	0.68 (0.10, 1.00)	93.0	17	0.45 (0.32, 0.58)	94.5	9	0.38 (0.31, 0.46)	78.4	0.542
Liver disease	3	0.24 (0.00, 0.72)	ID	12	0.34 (0.15, 0.54)	93.9	4	0.31 (0.12, 0.52)	0.0	0.595

*N* Number of studies, *P* Proportion, *CI* Confidence interval, *ID* Insufficient data, *NA* Not available, *HTN* Hypertension, *DM* Diabetes mellitus, *CVD* Cardiovascular disease, *CVA* Cerebrovascular accident, *CKD* Chronic kidney disease, *I*^2^ Index for between studies heterogeneity.

**Table 3:** The proportion estimates of underlying medical diseases and associated COVID-19 severity and mortality by gender distribution.

	Male dominant studies (≤ 50%)			Female dominant studies (> 50%)			*p* value
	N	P (95% CI)	I^2^	N	P (95% CI)	I^2^	
**Comorbidities**							
One comorbidity	57	0.51 (0.44, 0.57)	99.5	17	0.53 (0.40, 0.65)	98.4	0.728
Multiple comorbidities	8	0.27 (0.15, 0.40)	99.8	3	0.33 (0.16, 0.54)	ID	0.617
HTN	68	0.33 (0.28, 0.38)	99.1	23	0.28 (0.22, 0.34)	99.0	0.175
DM	73	0.19 (0.16, 0.22)	98.4	27	0.16 (0.12, 0.20)	98.7	0.112
CVD	70	0.16 (0.13, 0.20)	99.0	26	0.14 (0.08, 0.22)	99.6	0.498
Obesity	18	0.26 (0.17, 0.35)	99.5	6	0.22 (0.08, 0.40)	99.8	0.957
CVA	24	0.05 (0.03, 0.06)	88.4	9	0.04 (0.02, 0.06)	88.4	0.483
Lung disease	65	0.09 (0.07, 0.11)	97.4	22	0.08 (0.05, 0.12)	98.9	0.793
Cancer/malignancy	44	0.06 (0.04, 0.07)	96.8	16	0.04 (0.01, 0.07)	98.8	0.478
CKD/other renal disease	43	0.07 (0.05, 0.10)	98.8	17	0.04 (0.02, 0.06)	97.9	0.091
Liver disease	29	0.06 (0.03, 0.09)	98.6	16	0.03 (0.01, 0.06)	97.0	0.508
**Severity**							
One comorbidity	30	0.40 (0.35, 0.45)	96.8	8	0.46 (0.34, 0.58)	84.5	0.250
No comorbidity	30	0.17 (0.14, 0.21)	95.4	8	0.27 (0.13, 0.43)	94.4	0.682
Multiple comorbidities	7	0.38 (0.25, 0.53)	98.5	1	1.00 (0.63, 1.00)	ID	0.154
HTN	43	0.38 (0.33, 0.43)	92.4	15	0.44 (0.37, 0.52)	86.5	0.288
DM	42	0.41 (0.36, 0.46)	86.3	18	0.48 (0.38, 0.57)	89.7	0.414
CVD	41	0.43 (0.36, 0.50)	89.2	16	0.47 (0.37, 0.58)	86.2	0.900
Obesity	12	0.32 (0.24, 0.40)	94.5	4	0.36 (0.19, 0.55)	95.5	0.472
CVA	15	0.39 (0.28, 0.50)	64.9	6	0.54 (0.35, 0.73)	70.8	0.293
Lung disease	37	0.41 (0.34, 0.49)	85.0	15	0.42 (0.29, 0.56)	79.7	0.517
Cancer/malignancy	23	0.34 (0.24, 0.45)	81.5	11	0.44 (0.28, 0.60)	54.5	0.809
CKD/other renal disease	25	0.40 (0.31, 0.49)	80.0	9	0.76 (0.45, 0.98)	88.7	< 0.001
Liver disease	18	0.23 (0.12, 0.35)	58.5	11	0.27 (0.14, 0.43)	41.5	0.922
**Mortality**							
One comorbidity	24	0.29 (0.22, 0.36)	98.9	9	0.24 (0.15, 0.34)	94.3	0.514
No comorbidity	24	0.10 (0.06, 0.15)	98.7	9	0.01 (0.00, 0.03)	48.1	0.160
Multiple comorbidities	6	0.19 (0.14, 0.25)	96.4	1	0.26 (0.18, 0.36)	ID	0.804
HTN	31	0.33 (0.28, 0.38)	96.4	12	0.26 (0.17, 0.37)	98.0	0.098
DM	35	0.35 (0.29, 0.41)	96.7	15	0.29 (0.19, 0.39)	96.4	0.189
CVD	35	0.43 (0.38, 0.49)	95.6	14	0.36 (0.23, 0.49)	95.0	0.151
Obesity	8	0.32 (0.22, 0.43)	96.8	4	0.17 (0.08, 0.28)	87.9	0.205
CVA	11	0.47 (0.32, 0.62)	80.9	4	0.39 (0.08, 0.73)	74.9	0.870
Lung disease	31	0.37 (0.29, 0.44)	91.8	10	0.26 (0.17, 0.36)	84.4	0.410
Cancer/malignancy	22	0.36 (0.27, 0.45)	86.7	7	0.24 (0.16, 0.34)	25.5	0.334
CKD/other renal disease	23	0.44 (0.36, 0.53)	93.1	10	0.44 (0.23, 0.66)	96.4	0.565
Liver disease	13	0.29 (0.13, 0.48)	93.7	6	0.38 (0.07, 0.75)	72.0	0.803

*N* Number of studies, *P* Proportion, *CI* Confidence interval, ID Insufficient data, *HTN* Hypertension, *DM* Diabetes mellitus, *CVD* Cardiovascular disease, *CVA* Cerebrovascular accident, *CKD* Chronic kidney disease, *I*^2^ Index for between studies heterogeneity.

### Severity of COVID-19 patients within each comorbidity

**Figure 3:**
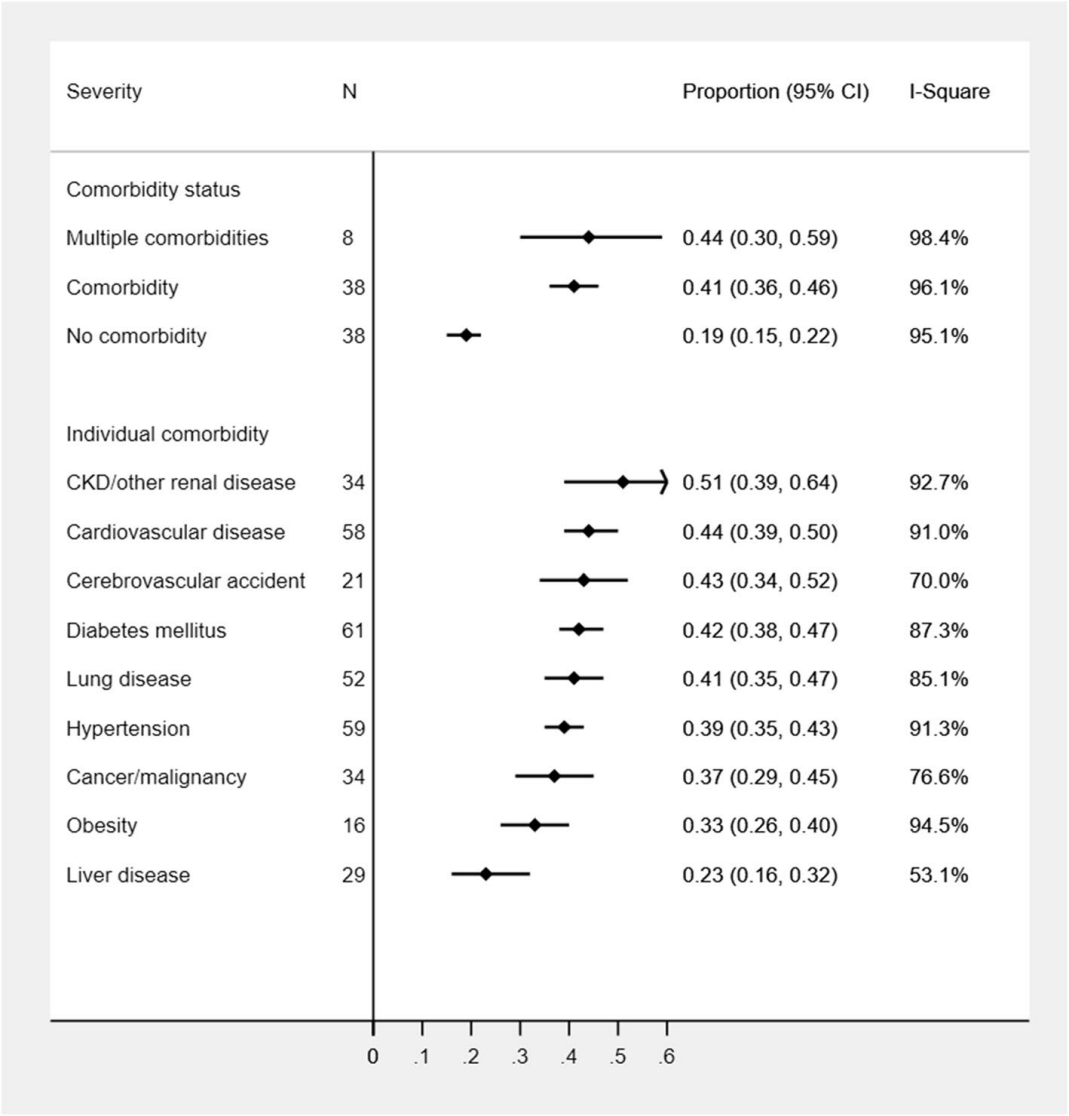
Proportion of COVID-19 severity among patients with underlying medical comorbidities.

The disease severity was highly prevalent in CKD patients (51%; 95%CI: 39–64%) whereas a similar level of severity of COVID-19 cases was observed in CVD (44%), CVA (43%), DM (42%), and lung disease (42%) conditions followed by HTN (39%) and malignancy (37%) conditions. The least proportion of disease severity was estimated in liver disease (23%) and obese (33%) patients. COVID-19 severity remained similar in patients with at least one comorbidity (41%) or multiple comorbidities (44%) but substantially higher than COVID-19 cases without any comorbidity (19%) (Fig. 3). Other than liver disease and obesity, COVID-19 severity (range: 40–68%) was found to be highest among patients with any comorbidities in Asian studies compared to the studies from other geographic locations (additional file: Supplementary Table 5). The most common cause of the severity of COVID-19 was CKD (68%; 95%CI: 46–87%) in Asian studies, liver disease (50%; 95%CI: 1–99%) in European and Latin American studies, and malignancy in the US studies (38%; 95%CI: 18–60%). Patients with medical conditions such as CVD, lung disease, and DM were associated with a similar level of severity in Asian studies (50–55%) (Table 2). Age and gender differences in COVID-19 severity according to comorbidities were not observed (additional file: Supplementary Table 6, Tables 2, 3). However, female dominant studies tended to show more severe cases among patients with HTN (44% vs. 38%), DM (48% vs. 41%), CVA (54% vs. 39%), and CKD (76% vs. 40%) compared to male-dominant studies (Table 3).

### Mortality of COVID-19 patients within each comorbidity

**Figure 4:**
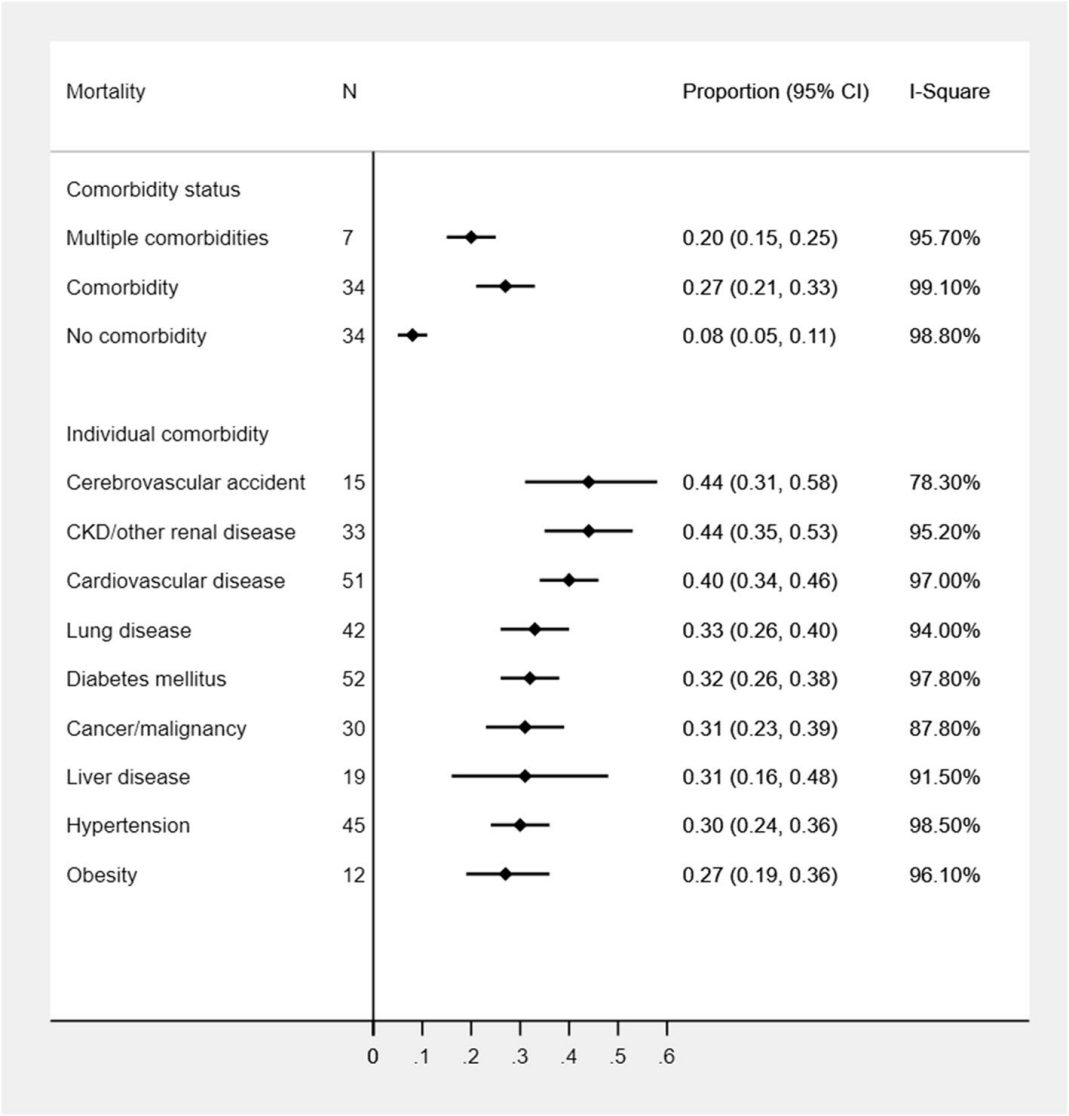
Proportion of mortality among COVID-19 patients with underlying medical comorbidities.

Compared to COVID-19 patients without any preexisting condition, the overall mortality proportion was much higher among patients with CVA (44%, 95% CI: 31%, 58%), CKD (44%, 95% CI: 35%, 53%), and CVD (40%, 95% CI: 34%, 46%). The other underlying conditions were associated with similar levels of mortality (range: 30–33%) except for obesity (27%) (Fig. 4). In Latin American studies, the highest proportion of mortality was observed in CKD (81%) and liver disease (65%) patients. The mortality proportion remained similar in other conditions (51–55%) in Latin American studies but higher than studies from other geographic regions. Similarly, COVID-19 coexisted with CKD (60%) or liver disease (49%) had the highest mortality in European studies as well. European patients with CVD (44%), malignancy (44%), DM (41%), lung disease (40%), obesity (37%), and HTN (36%) had a higher mortality rate compared to other studies from the US or Asia. The highest mortality was observed in patients with CVA (48%) followed by CKD (38%) and CVD (36%) in Asian studies whereas CVD (40%) and CVA (39%) had the highest mortality in the US studies (Table 1). Significant geographic differences in mortality proportions associated with underlying medical conditions were also observed (Table 1 and additional file: Supplementary Table 5). Older adults without any comorbidities had a 14% mortality rate (95%CI: 3–30%) with 10% (95%CI: 6–15%) in predominantly male studies without any medical conditions. An increasing trend in mortality proportion was observed with higher age groups in COVID-19 patients with CVD, malignancy, and one comorbidity whereas a decreasing trend in mortality proportion was noticed in CKD patients (Table 2). Compared to female-dominant studies, male-dominant studies had a high rate of mortality among patients with any comorbid conditions other than CKD and liver disease. However, gender differences in mortality within each comorbidity were not found to be statistically significant (Table 3).

## Discussion

In our meta-analysis, HTN, obesity, DM, and CVD were more frequently observed in COVID-19 patients whereas mortality was more frequently observed in COVID-19 patients with CVD, CVA, and CKD. The presence of CVD and their most prominent risk factors such as HTN and DM have been shown to be associated with COVID-19 disease^11,38^ because SARS-CoV-2 binds to the angiotensin-converting enzyme 2 (ACE2) receptor by interaction with viral spike protein which allows the virus to enter into host cells^39–41^. Furthermore, several medications for the treatment of COVID-19 may yield cardiovascular adverse events, and COVID-19 may also directly cause CVD or aggravate underlying CVD^40,42,43^. Interaction of SARS-CoV-2 with the renin–angiotensin–aldosterone system (RAAS) may likely to observe in HTN and DM patients reflecting as the most prevalent conditions in COVID-19 patients^9^. Furthermore, immunocompromised patients are at high risk for developing any infectious diseases and immune dysfunction has been linked with HTN, DM, obesity, CVD, lung disease, and CKD indicating these medical conditions as more common in COVID-19 patients as well^42–44^. Patients with CVD, CKD, and a history of CVA have been observed at high risk for mortality in COVID-19 in our study. These conditions share common risk factors including increased allostatic load, and COVID-19 may either induce or aggravate these conditions by impacting ACE2 receptor, RAAS function, immune, hemostatic and nervous system damage, and major physiological systems^40,45^. Our study suggests that COVID-19 patients with chronic diseases regardless of geographic location should be given proper health care attention and prioritized for vaccination.

The highest mortality among all comorbidities was observed in European studies compared to the US and Asian studies despite lower prevalence of comorbidities compared to the US studies and lower severity prevalence in each comorbidity compared to Asian studies. This mostly occurred due to more elderly and male patients compounded with the second highest proportions of underlying medical conditions and disease severity in some comorbidities in European studies. In addition, an early hit of the pandemic in European regions especially in the elderly population might have also created a surge in these populations. In contrast, a single study from South Africa yielded the lowest mortality rate even with high proportions of DM (40.4%) and HTN (42%) patients. This could be due to the inclusion of predominantly females in the study compared to other region studies. Older age, male gender, and presence of at least one comorbidity have been shown to be independently associated with mortality in COVID-19 patients^9^. In another meta-analysis^10^, the mortality rate in COVID-19 has also been reported to be highest in the European region compared to the US or other regions. Our meta-analysis also estimated the highest rate of mortality in Latin American studies despite having younger patients than studies from other regions. Although the specific reason for this finding is yet not clear, a limited number of studies from Latin American regions might have amplified the proportion estimates of mortality among different comorbid conditions. Other potential reasons for having high mortality in Latin American countries could be the differential approaches to COVID-19 testing and timing and implementation of mitigation strategies compared to other geographic regions along with resource constrained health systems, allocation strategies of resources, inequality and poverty, and internal political dynamics^30,46^. Similar to another meta-analysis^10^, our study also confirmed the lower rate of mortality in the Asian region and a South African study than the European and American regions. It appears that the varying immune profile of individuals from different racial groups can modulate their risk of disease infection and autoimmunity and may respond differently to novel infections^47^. Preliminary evidence showed a trend towards the slower spread of COVID-19 in countries where the national Bacillus Calmette–Guérin vaccine policy is current for all^48^. Based on distributions of age and gender with differential prevalence of comorbid conditions associated with disease outcomes specific to geographic regions, we suggest the joint evaluation of country-specific demographic and comorbid factors for stratifying risk for COVID-19 disease severity and mortality.

Our study estimated the highest proportion of all comorbidities in US studies compared to studies from other countries. The prevalence of comorbid conditions have been reported to be higher in the overall US population which may be reflecting the higher prevalence of each comorbid condition in COVID-19 patients as well^49–51^. COVID-19 severity in the US remained lower than Asian and European studies even with high proportions of HTN and multiple comorbidities. This empirical observation indirectly suggests that RAAS inhibitors may not adversely impact COVID-19 patients with underlying medical conditions, although this needs further validation^39,52^. The low rate of mortality within each comorbidity in the US studies even with the high prevalence of underlying disease conditions is likely due to optimal management of COVID-19 cases with underlying disorders, timely and strategic implementation of mitigation strategies, avoiding unnecessary medication use, and proper reporting of public health practices^30^. Our stratified analyses clearly showed that higher COVID-19 severity was more likely to occur in all underlying medical conditions in the Asian region compared to other regions. Although a direct comparison of severity among each of the comorbid conditions is not available in COVID-19 across geographic locations, socio-demographic, cultural, behavioral, and biological factors might explain the differential prevalence of the disease severity in Asian studies^47,53^. Our study suggests developing COVID-19 management policies specific to geographic regions. For example, some regions need proper implementation of mitigation strategies and vaccination policy to minimize COVID-19 spread among at-risk individuals while some regions require adequate resources with proper guidelines, and management strategies for minimizing disease severity.

This study has confirmed higher mortality in older adults (> 65 years) with all the underlying medical diseases except for CKD patients. This could be due to the inclusion of Latin American studies involving the youngest age patients and demonstrating a higher rate of mortality in CKD and obese patients. Mortality risk in older adults with comorbidities was also confirmed in other studies^29,54,55^. Similar to our study, a study also confirmed that older adults with underlying diseases including DM, HTN, CVD, liver diseases, and malignancy have been more likely to develop a critical illness^56^. Our meta-analysis also confirmed that there was no substantial difference in the burden of comorbidities between male or female-dominant studies. In consistent with a single-center retrospective study^29^ and another meta-analysis^57^, we also observed higher rates of disease severity with most of the underlying medical conditions in female-dominant studies while higher mortality rates were observed in male-dominant studies. Gender differences in mortality rates in all underlying medical conditions could be due to the sex hormones and sex chromosome genes associated with different immune responses^58,59^. Like we observed geographical differences in COVID-19 severity and mortality, the geographical differences in COVID-19 therapeutic practices have also been observed^60^. COVID-19 prevention and treatment strategies should be effectively implemented to elderly population and individuals with underlying disease conditions.

We have some limitations in our study. Although we have used random effects models to estimate proportions, high heterogeneity across the studies and publication biases in few subgroup analyses might produce biased estimates. Given the variations in study populations, we estimated prevalence estimates within each comorbidity stratified by geographic regions. However, the variation in racial/ethnic and socioeconomic populations might further yield heterogeneity in the proportion estimates. Limited studies from Africa and Latin America may produce higher estimates in these subpopulations. Furthermore, we could not explore the regional differences in disease outcomes in Asian countries due to limited studies from South and West Asia. Since the majority of the studies included in our study were retrospective and conducted in different countries, missing, misclassification, and variation in reporting of medical conditions may be present. While our study is the most update-to-date and comprehensive, our restriction to studies with only English language and upcoming evidence as COVID-19 is evolving might impact the findings of our study. Despite these limitations, to the best of our knowledge, our study is the first comprehensive systematic review and meta-analysis that produced estimates of major underlying medical conditions with COVID-19, and COVID-19 severity and mortality within each comorbidity by including a large number of high-quality studies from the US, Europe, Asia, and other countries with large sample sizes. Our methodological rigor and stratified analysis of the study population by geographic regions yielded reliable and regional specific findings to develop an effective control policy for this COVID-19 pandemic.

## Conclusions

In conclusion, we observed high prevalence estimates of major comorbid conditions in US studies. Among patients with any comorbidities, the highest disease severity was estimated in Asian region while the highest mortality in European and Latin American regions. The mortality among those with underlying medical diseases was estimated to be high in mostly elderly and predominantly male patients with considerable mortality in older patients without any comorbidities. COVID-19 severity and mortality were highly variable based on medical conditions specific to geographic regions. These findings clearly suggest that country-specific comorbidities should ideally be used to evaluate and stratify risk for the COVID-19 disease severity and mortality. We suggest developing policies across the globe with particular attention to specific geographic/ethnic populations such as avoiding COVID-19 spread to individuals with underlying medical conditions especially in the US population, protecting elderly male and sick individuals from SARS-CoV-2 infection mainly from European regions, and managing life-threatening COVID-19 complications particularly in Asian patients. In addition, at-risk individuals according to geographic regions should be given a priority for vaccination. Our study prioritizes comorbid conditions associated with the disease severity and mortality and provides insights for targeted risk stratification and effective control management of this pandemic.

## Supplementary information

Supplementary information

## Data Availability

The dataset for the current study is available from the corresponding author on reasonable request.

## Abbreviations

COVID-19: Coronavirus disease 2019
SARS-CoV-2: Severe acute respiratory syndrome associated coronavirus 2
SARS-CoV-1: Severe acute respiratory syndrome associated coronavirus 1
WHO: World health organization
DM: Diabetes mellitus
HTN: Hypertension
CKD: Chronic kidney disease
COPD: Chronic obstructive pulmonary disease
CVD: Cardiovascular disease
CVA: Cerebrovascular accident
PRISMA: Preferred reporting in systematic reviews and meta-analyses
SAMBR: Statistical analysis and methods in biomedical research
ICU: Intensive care unit
MINORS: Methodological index for non-randomized studies
CI: Confidence interval
ACE2: Angiotensin-converting enzyme 2
RAAS: Renin–angiotensin–aldosterone system
