# Supplementary material for: A systematic review and meta-analysis of geographic differences in comorbidities and associated severity and mortality among individuals with COVID-19
Source: Sci Rep. 2021 Apr 20;11:8562. doi: 10.1038/s41598-021-88130-w (PMC8058064; doi:10.1038/s41598-021-88130-w)
Supplement: Supplementary file 1 — Supplementary information [file 41598_2021_88130_MOESM1_ESM.pdf]

A systematic review and meta-analysis of geographic differences in comorbidities and associated severity and mortality among individuals with COVID-19

Bhaskar Thakur[1], Pallavi Dubey[2], Joseph Benitez[3], Joshua P. Torres[3], Sireesha Reddy[2], Navkiran Shokar[4], Koko Aung[5], Debabrata Mukherjee[5], Alok K. Dwivedi[1,3,6].

[1]Division of Biostatistics and Epidemiology, Department of Molecular and Translational Medicine, Paul L. Foster School of Medicine, Texas Tech University Health Sciences Center El Paso, 5001 El Paso Drive, TX, 79905, USA.

[2]Department of Obstetrics & Gynecology, Paul L. Foster School of Medicine, Texas Tech University Health Sciences Center El Paso, 4800 Alberta Avenue, El Paso, TX, 79905, USA.

[3]Graduate School of Biomedical Sciences, Texas Tech University Health Sciences Center El Paso, 5001 El Paso Drive, TX, 79905, USA.

[4]Department of Family and Community Medicine, Paul L. Foster School of Medicine, Texas Tech University Health Sciences Center El Paso, 9849 Kenworthy St., TX, 79924, USA.

[5] Department of Internal Medicine, Texas Tech University Health Science Center El Paso, Paul L. Foster School of Medicine, 4800 Alberta Ave, El Paso, Texas, 79905, USA.

[6] Biostatistics and Epidemiological Consulting Lab, Texas Tech University Health Sciences Center El Paso, 5001 El Paso Drive, TX, 79905, USA.

Corresponding Author:

Alok Kumar Dwivedi, Department of Molecular and Translational Medicine, Paul L. Foster School of Medicine, Texas Tech University Health Sciences Center El Paso, Medical Science Building, 5001 El Paso Drive, El Paso Texas 79905, USA

Email: alok.dwivedi@ttuhsc.edu

Tel: 9152154177

Comorbidities

We collected status of the comorbidities (number of subjects without any comorbidity, number of subjects with one comorbidity, number of subjects with multiple comorbidities), the type of comorbidity that included hypertension (HTN), diabetes mellitus (DM), cardiovascular disease (CVD), obesity (OB), cerebrovascular accident (CVA), lung disease, cancer/malignancy, either of chronic or acute kidney disease (CKD) and liver related disease.

In our study, the HTN includes isolated hypertension, pulmonary hypertension, and high blood pressure. The CVD includes heart failure, atrial fibrillation, coronary heart disease, congestive heart failure, cardiac insufficiency, subvalvular aortic stenosis, myocardial infarction and other ischemic heart diseases. Cerebrovascular disease includes a history of all types of strokes and cerebrovascular accident. Chronic lung diseases involves chronic lung disease , asthma, chronic obstructive pulmonary disease , chronic respiratory disease, small airway obstruction syndrome, chronic lower respiratory disease, environmental lung disease, interstitial lung disease, chronic respiratory failure, diaphragm palsy, pulmonary vascular disease, tuberculosis, bronchitis, emphysema, and other chronic lung diseases. Cancer/malignancy contains solid malignant tumors, hematological malignant disease, and any other form of cancers or malignancies. CKD/ other renal disease consists of acute kidney injury, chronic kidney disease, end stage renal disease, and other renal diseases.

Primary outcomes

The primary outcomes in this study were COVID-19 severity and mortality. Any COVID-19 patients who had one of the following criteria: (a) admitted to the intensive care unit (ICU), (b) required mechanical ventilation support, (c) required mechanical shock/life support, (d) developed sepsis or septic shock, or (e) poor prognosis or labeled as critically ill by the attending physicians, were classified as severe COVID-19. Any study who reported death as the outcome for COVID-19 patients regardless of any underlying comorbidities was considered as mortality event due to COVID-19.

Comparing variables

The primary variables of interest for comparing outcomes were geographic location, age groups, and gender. The geographic location was grouped into Asia (China, India, Iran, Oman, South Korea), Europe (Denmark, France, Germany, Italy, Netherlands, Poland, Spain, Switzerland, Turkey, UK, and Italy), Latin America (Brazil and Mexico), South Africa, and USA. Based on the distribution of average age, the age was categorized into ≤ 50 years, 51 – 65 years, and > 65 years. Similarly, the percentage of the female population was divided into two groups as ≤ 50%: male dominant and > 50%: female dominant.

Quality Assessment

Only seven out of the first eight items (criteria to assess the non-randomized and non-comparative studies) of MINORS were used in this study. Item number five (unbiased assessment of the study endpoint) was not used as the study endpoints were straight forward and the blind evaluation was not needed. Since the majority of the included studies were cross-sectional conducted on hospitalization of COVID-19 patients, we did not report any loss to follow up. A score from 0 to 2 was given for all the seven items according to whether the item related attributes were reported or not as well as adequate or not [0 (unreported), 1 (reported but inadequate) or 2 (reported and adequate)]. The total score assigned to each study was categorized for the quality assessment which follows: 0–2, very low quality; 3–6, low quality; 7–10, fair quality; and 11–14, high quality. In addition, we also evaluated publication bias for the primary outcomes within each comorbidity using Egger's test.

Supplementary Table 1

Characteristics and quality assessment score of included studies

Study	Country	No. of patients	No. of Female (%)	Age, mean ± SD /median (IQR)	Comorbidity	Multiple morbidities	Severity	Mortality	Hospitalization status	Quality assessment
Wang, L et al. [1]	China	339	173 (51)	71 ± 8	206	NA	NA	NA	Hospitalized	12
Cao, J et al. [2]	China	102	49 (48)	54 (37, 67)	47	NA	NA	17	Hospitalized	13
Chen, T et al. [3]	China	274	103 (37·6)	62 (44, 70)	133	NA	NA	113	Hospitalized	12
Deng, Y et al. [4]	China	225	101 (44·9)	69 (62, 74)	127	NA	NA	109	Hospitalized	12
Yuan, M et al. [5]	China	27	15 (55·6)	60 (47, 69)	13	NA	NA	10	Hospitalized	10
Wu, C et al. [6]	China	201	73 (36·3)	51 (43, 60)	66	NA	NA	NA	Hospitalized	12
Li, K et al. [7]	China	83	39 (47)	45·5 ± 12·3	15	NA	25	NA	Hospitalized	12
Yang, X et al.[8]	China	52	17 (32·7)	59·7 ± 13·3	21	NA	NA	32	Hospitalized	11
Zhang, JJ et al. [9]	China	140	69 (49·3)	57 (25, 87)	90	NA	58	NA	Hospitalized	13
Wang, D et al. [10]	China	138	63 (45·7)	56 (42, 68)	64	NA	36	NA	Hospitalized	13
Huang, C et al. [11]	China	41	11 (26·8)	49 (41, 58)	13	NA	13	NA	Hospitalized	12
Wang, Z et al. [12]	China	69	37 (53·6)	42 (35, 62)	25	NA	NA	NA	Hospitalized	12
Guan, WJ et al. [13]	China	1590	686 (43·1)	48·9 ± 16·3	399	52	254	50	Hospitalized	13
Chen, T et al. [14]	China	55	21 (38·2)	74 (65, 91)	35	NA	NA	19	Hospitalized	11

Chen, Q et al. [15]	China	145	66 (45·5)	47·5 ± 14·6	75	NA	43	NA	Hospitalized	12
Zhang, G et al. [16]	China	221	113 (51·1)	55 (39, 67)	78	NA	55	NA	Hospitalized	12
Zhang, R et al. [17]	China	120	77 (64·2)	45·4 ± 15·6	32	NA	30	NA	Hospitalized	12
Nikpouraghdam, M et al. [18]	Iran	2964	1009 (34)	56 (46, 65)	323	NA	NA	239	Hospitalized	9
Li, Y et al.[19]	China	54	20 (37)	61·8 ± 14·5	30	NA	54	NA	Hospitalized	11
Zhou, F et al. [20]	China	191	73 (38·2)	56 (46, 67)	91	NA	NA	54	Hospitalized	12
He, R et al. [21]	China	204	125 (61·3)	49 (34, 62)	57	NA	NA	NA	Hospitalized	12
Yang, AP et al. [22]	China	93	37 (39·8)	46·4 ± 17·6	50	NA	24	NA	Hospitalized	11
Borobia, AM et al. [23]	Spain	2226	1152 (51·8)	61 (46, 78)	1747	NA	NA	460	Hospitalized	13
Zhang, C et al. [24]	China	80	47 (58·8)	51·2 ± 17·5	56	NA	NA	NA	Hospitalized	11
Zhao, J et al. [25]	China	29	15 (51·7)	56 (32, 66)	19	8	21	1	Hospitalized	11
Yao, Q et al. [26]	China	108	65 (60·2)	52 (37, 58)	25	NA	25	12	Hospitalized	13
Kayem, G et al.[27]	France	617	100 (100)	NA	NA	NA	NA	NA	Hospitalized	9
Tambe, MP et al.[28]	India	197	107 (54·3)	45·8 ± 17·3	93	NA	NA	58	Hospitalized	13
Yang, Q et al. [29]	China	136	70 (51·5)	56 (44, 64)	NA	NA	NA	NA	Hospitalized	12
Şenkal, N et al.[30]	Turkey	611	248 (40·6)	57 ± 15	NA	NA	NA	NA	Hospitalized	13
Shi, S et al.[31]	China	416	211 (50·7)	64 (21, 95)	NA	NA	NA	NA	Hospitalized	12
Mani, VR et al.[32]	USA	184	73 (39·7)	64·7 ± 14·9	NA	NA	NA	NA	Hospitalized	12
Li, M et al.[33]	China	83	49 (59)	43 (32, 62)	42	NA	18	6	Hospitalized	11
Salacup, G et al.[34]	USA	242	119 (49·2)	66 (58, 76)	NA	NA	NA	NA	Hospitalized	12

Shi, S et al.[35]	China	671	322 (48)	63 (50, 72)	NA	NA	NA	NA	Hospitalized	13
Kalyanaraman Marcello, R. et al.[36]	USA	6248	2396 (38·3)	61 (50, 73)	NA	NA	NA	NA	Hospitalized	13
Israelsen, SB et al.[37]	Denmark	175	90 (51·4)	71 (55, 81)	125	NA	NA	NA	Hospitalized	12
Pellaud, C et al.[38]	Switzerland	196	77 (39·3)	70 (60 , 80)	162	NA	49	NA	Hospitalized	12
Gregoriano, C et al.[39]	Switzerland	99	37 (37·4)	67 (56, 76)	NA	NA	NA	NA	Hospitalized	11
Argenziano, MG et al.[40]	USA	1000	65 (6·5)	55 (40, 69)	918	NA	386	NA	Hospitalized	13
Borghesi, A et al.[41]	Italy	302	108 (35·8)	67 (57, 77)	NA	NA	NA	NA	Hospitalized	12
Toussie, D et al.[42]	USA	338	128 (37·9)	39 (31, 45)	NA	NA	NA	NA	Hospitalized	12
Ortiz-Brizuela, E et al.[43]	Mexico	140	55 (39·3)	78 (70, 88)	NA	NA	NA	NA	Hospitalized	13
Aggarwal, A et al.[44]	India	32	13 (40·6)	55 (46, 60)	22	11	24	NA	Hospitalized	11
Javanian, M et al.[45]	Iran	100	49 (49)	60·1 ± 13·9	50	NA	NA	NA	Hospitalized	12
Huang, Q et al.[46]	China	54	26 (48·1)	41 (31, 51)	18	NA	3	NA	Hospitalized	11
Pelayo, J et al. [47]	USA	223	50 (22·4)	65·9 ± 14·9	NA	NA	NA	NA	Hospitalized	12
Alkundi, A et al.[48]	UK	232	87 (37·5)	70·5 ± 15·7	NA	NA	NA	NA	Hospitalized	12
Qin, C et al. [49]	China	1875	930 (49·6)	63 (51, 70)	NA	NA	NA	NA	Hospitalized	13
Chen, Y et al.[50]	China	904	483 (53·4)	56(39, 67)	NA	NA	NA	NA	Hospitalized	13
Khamis, F et al. [51]	Oman	63	10 (15·9)	48 ± 16	32	NA	NA	NA	Hospitalized	12
Gayam, V et al.[52]	USA	408	177 (43·4)	67 (56, 76)	NA	NA	NA	NA	Hospitalized	12
Shi, Q et al.[53]	China	306	156 (51)	64 (56, 72)	NA	NA	NA	NA	Hospitalized	12
Nowak, B et al.[54]	Poland	169	82 (48·5)	63·7 ± 19·6	137	NA	NA	NA	Hospitalized	12

Rottoli, M et al.[55]	Italy	482	163 (33·8)	66·2 ± 16·8	NA	NA	NA	NA	Hospitalized	12
Huang, Y et al.[56]	China	1255	589 (46·9)	64(52, 70)	791	NA	236	NA	Hospitalized	9
Li, T et al.[57]	China	312	125 (40·1)	69·2 ± 7·3	241	NA	105	NA	Hospitalized	12
Zhou, X et al.[58]	China	110	50 (45·5)	57·7 ± 14·2	NA	NA	NA	NA	Hospitalized	12
Wu, F et al.[59]	China	1048	457 (43·6)	63·5	382	NA	NA	NA	Hospitalized and non-hospitalized	12
Xu, J et al.[60]	China	239	96 (40·2)	62·5 ± 13·3	162	NA	NA	NA	Hospitalized	11
Buckner, FS et al.[61]	USA	105	52 (49·5)	69(23, 97)	98	NA	51	NA	Hospitalized	12
Wan, S et al.[62]	China	135	63 (46·7)	47(36, 55)	43	NA	40	NA	Hospitalized	13
Li, J et al. [63]	China	94	43 (45·7)	58·7 ± 16	39	NA	NA	13	Hospitalized	10
Cao, M et al.[64]	China	198	97 (49)	50·1 ± 16·3	69	NA	19	NA	Hospitalized	12
Sun, L et al. [65]	China	55	24 (43·6)	44 (34, 56)	18	NA	15	NA	Hospitalized	11
Huang, R et al.[66]	China	202	86 (42·6)	44 (33, 54)	55	NA	23	NA	Hospitalized	12
Labenz, C et al.[67]	Germany	42	13 (31)	68 (55, 75)	NA	NA	NA	NA	Hospitalized	11
Zhang, JJ et al. [68]	China	289	135 (46·7)	57 (22, 88)	169	NA	127	49	Hospitalized	13
Pérez, FM et al.[69]	Spain	96	33 (34·4)	63 ± 17	60	NA	17	NA	Hospitalized	10
Wang, L et al. [70]	China	116	49 (42·2)	54 (38–69)	51	NA	NA	7	Hospitalized	13
Wang, D et al.[71]	China	143	70 (49)	58 (39–67)	50	NA	71	NA	Hospitalized	12
Güner, R et al. [72]	Turkey	222	90 (73·8)	50·6 ± 16·5	92	NA	50	NA	Hospitalized	12
Zhou, Y et al.[73]	China	304	166 (54·6)	61·5 ± 13·3	NA	NA	NA	NA	Hospitalized	12
Feng, Y et al.[74]	China	476	205 (43·1)	53 (40, 64)	205	NA	124	NA	Hospitalized	12

Brill, SE et al.[75]	UK	450	178 (39·6)	72 (56, 83)	NA	NA	NA	NA	Hospitalized	12
Shah, P et al.[76]	USA	522	304 (58·2)	63 (50 , 72)	NA	NA	NA	NA	Hospitalized	13
Lagi, F et al.[77]	Italy	84	29 (34·5)	62 (51, 72)	49	NA	NA	NA	Hospitalized	12
Yang, Q et al.[78]	China	226	113 (50)	49·9 ± 15·3	NA	NA	NA	NA	Hospitalized	12
Ji, W et al.[79]	South Korea	7341	4371 (59·5)	47·1 ± 19·0	NA	NA	NA	NA	Hospitalized and non-hospitalized	13
Lee, JY et al.[80]	South Korea	694	482 (69·5)	55·9 ± 17·3	NA	NA	NA	NA	Hospitalized	13
Shahriarirad, R et al. [81]	Iran	113	42 (37·2)	53·7 ± 16·6	44	NA	NA	NA	Hospitalized	12
Baqui, P et al.[82]	Brazil	7371	3081 (41·8)	58·3 ± 16·4	NA	NA	NA	NA	Hospitalized	12
Asfahan, S et al.[83]	India	20812	NA	NA	5276	NA	NA	504	Hospitalized and non-hospitalized	12
Petrilli, CM et al.[84]	USA	2729	1057 (38·7)	63 (51, 74)	2176	NA	990	NA	Hospitalized	14
Mendy, A et al.[85]	USA	689	324 (47)	49·5 ± 33·1	NA	NA	NA	NA	Hospitalized	12
Masetti, C et al. [86]	Italy	229	81 (35·4)	60·7 ± 14·2	124	NA	NA	33	Hospitalized	12
Davies, MA et al. [87]	South Africa	2978	1848 (62·1)	NA	NA	NA	NA	NA	Hospitalized	13
Huang, J et al.[88]	China	299	139 (46·5)	53·4 ± 16·7	99	NA	NA	16	Hospitalized	12
Kammar-Garcia, A et al. [89]	Mexico	13842	5853 (42·3)	46·6 ± 15·6	6270	1282	4098	1305	Hospitalized	12
Ye, C et al.[90]	China	856	417 (48·7)	46 (35-56)	242	63	154	1	Hospitalized	13
Bonetti, G et al.[91]	Italy	144	49 (34)	70	92	NA	NA	70	Hospitalized	12
Ponziani, FR et al.[92]	Italy	515	192 (37·3)	65 (53, 77)	350	32	77	NA	Hospitalized	13
Klang, E et al.[93]	USA	3406	1445 (42·4)	65·94	3126	NA	NA	1136	Hospitalized	13

Sousa, GJB et al.[94]	Brazil	2070	1053 (50·9)	44 (34, 59)	NA	NA	NA	NA	Hospitalized	13
Brouns, SH et al.[95]	Netherlands	101	68 (67·3)	85·0 ± 8·1	NA	NA	NA	NA	Hospitalized	11
Lorente-Ros, A et al.[96]	Spain	707	264 (37·3)	66·6 ± 15·7	NA	NA	NA	NA	Hospitalized	14
Tatum, D et al.[97]	USA	125	68 (54·4)	58·7 ± 14·8	120	NA	NA	23	Hospitalized	13
Sabri, A et al.[98]	Iran	63	NA	54·1 ± 15·5	37	NA	NA	NA	Hospitalized	11
Cai, Q et al.[99]	China	383	200 (52·2)	49·2	NA	NA	NA	NA	Hospitalized	12
Pettit, NN et al.[100]	USA	238	125 (52·5)	58·5 ± 17	NA	NA	NA	NA	Hospitalized	12
Ji, D et al.[101]	China	208	91 (43·8)	44·0 ± 16·3	45	NA	40	NA	Hospitalized	12
Gidari, A et al.[102]	Italy	68	23 (33·8)	64 ± 14	NA	NA	NA	NA	Hospitalized	11
Du, RH et al.[103]	China	179	82 (45·8)	57·6 ± 13·7	NA	NA	NA	NA	Hospitalized	13
Sarin, SK et al. [104]	Asia	228	96 (42·1)	51·1	NA	NA	NA	NA	Hospitalized	12
Ebinger, JE et al.[105]	USA	442	186 (42·1)	52·7 ± 19·6	NA	NA	NA	NA	Hospitalized	12
Pagnesi, M et al.[106]	Italy	200	69 (34·5)	62 (55, 74)	NA	NA	NA	NA	Hospitalized	14
Rastad, H et al.[107]	Iran	2957	1368 (46·3)	54·8 ± 16·9	749	NA	NA	301	Hospitalized	13
van Gerwen, M et al. [108]	USA	3703	1654 (44·7)	56·8 ± 18·2	2398	322	525	616	Hospitalized	13
Zhou, Y et al.[109]	China	17	11 (64·7)	41·8	5	NA	5	NA	Hospitalized	11
Grasselli, G et al.[110]	Italy	3988	800 (20·1)	63 (56, 69)	2686	NA	NA	1926	Hospitalized	13
Xu, PP et al.[111]	China	703	321 (45·7)	46·1 ± 15·2	201	24	55	33	Hospitalized	13
Cen, Y et al.[112]	China	1007	514 (51)	61 (49 , 68)	364	NA	222	43	Hospitalized	14
Soares, RCM et al.[113]	Brazil	1152	494 (42·9)	NA	526	NA	NA	456	Hospitalized	10
Li, X et al.[114]	China	548	269 (49·1)	60 (48, 69)	NA	NA	NA	NA	Hospitalized	14

Gao, S et al. [115]	China	210	109 (51·9)	71 (67, 77)	159	NA	NA	35	Hospitalized	12
Urra, JM et al.[116]	Spain	172	68 (39·5)	59·1	NA	NA	NA	NA	Hospitalized	12
Rivera-Izquierdo, M et al.[117]	Spain	238	107 (45)	64·7 ± 15·4	135	NA	NA	61	Hospitalized	12
Deng, Q et al.[118]	China	112	55 (49·1)	65 (49, 71)	58	NA	NA	14	Hospitalized	12
Kim, DW et al.[119]	South Korea	9148	5592 (61·0)	NA	NA	NA	NA	NA	Hospitalized and non-hospitalized	13
Hewitt, J et al.[120]	UK & Italy	1564	661 (42·3)	74 (61 , 83)	NA	NA	NA	NA	Hospitalized	14

NA not available.

Supplementary Table 2

Quality appraisal of included studies according to MINORS assessment

**Study**	**Clearly stated aim**	**Inclusion of consecutive patients**	**Prospective collection of data**	**Endpoints appropriate to the aim of the study**	**Unbiased assessment of the study endpoint**	**Follow-up period appropriate to the aim of the study**	**Loss to follow up less than 5%**	**Prospective Calculation of the study size**
Wang, L et al. [1]	2	2	1	2	0	2	2	1
Cao, J et al. [2]	2	2	2	2	0	2	2	1
Chen, T et al.[3]	2	2	1	2	0	2	2	1
Deng, Y et al. [4]	2	2	1	2	0	2	2	1
Yuan, M et al. [5]	2	2	1	2	0	2	1	0
Wu, C et al. [6]	2	2	1	2	0	2	2	1
Li, K et al. [7]	2	2	1	2	0	2	2	1
Yang, X et al.[8]	2	2	1	2	0	2	2	0
Zhang, JJ et al. [9]	2	2	2	2	0	2	2	1
Wang, D et al. [10]	2	2	2	2	0	2	2	1
Huang, C et al. [11]	2	2	2	2	0	2	2	0
Wang, Z et al. [12]	2	2	2	2	0	2	2	0
Guan, WJ et al. [13]	2	2	1	2	0	2	2	2

Chen, T et al. [14]	2	2	1	2	0	2	2	0
Chen, Q et al. [15]	2	2	1	2	0	2	2	1
Zhang, G et al.[16]	2	2	1	2	0	2	2	1
Zhang, R et al. [17]	2	2	1	2	0	2	2	1
Nikpouraghdam, M et al. [18]	2	2	1	2	0	0	0	2
Li, Y et al.[19]	2	2	1	2	0	2	2	0
Zhou, F et al.[20]	2	2	1	2	0	2	2	1
He, R et al. [36]	2	2	1	2	0	2	2	1
Yang, AP et al. [22]	2	2	1	2	0	2	2	0
Borobia, AM et al. [23]	2	2	2	2	0	1	2	2
Zhang, C et al. [24]	2	2	1	2	0	2	2	0
Zhao, J et al. [25]	2	2	1	2	0	2	2	0
Yao, Q et al. [26]	2	2	1	2	0	2	2	2
Kayem, G et al.[27]	2	2	1	2	0	0	0	2
Tambe, MP et al.[28]	2	2	2	2	0	2	2	1
Yang, Q et al. [29]	2	2	1	2	0	2	2	1
Şenkal, N et al.[30]	2	2	1	2	0	2	2	2
Shi, S et al.[31]	2	2	1	2	0	2	2	1
Mani, VR et al.[32]	2	2	1	2	0	2	2	1
Li, M et al.[33]	2	2	1	2	0	2	2	0
Salacup, G et al.[34]	2	2	1	2	0	2	2	1
Shi, S et al.[35]	2	2	1	2	0	2	2	2
Kalyanaraman Marcello, R. et al.[36]	2	2	1	2	0	2	2	2
Israelsen, SB et al.[37]	2	2	1	2	0	2	2	1
Pellaud, C et al.[38]	2	2	1	2	0	2	2	1
Gregoriano, C et al.[39]	2	2	1	2	0	2	2	0
Argenziano, MG et al.[40]	2	2	1	2	0	2	2	2
Borghesi, A et al.[41]	2	2	1	2	0	2	2	1
Toussie, D et al.[42]	2	2	1	2	0	2	2	1

Ortiz-Brizuela, E et al.[43]	2	2	2	2	0	2	2	1
Aggarwal, A et al.[44]	2	2	1	2	0	2	2	0
Javanian, M et al.[45]	2	2	1	2	0	2	2	1
Huang, Q et al.[46]	2	2	1	2	0	2	2	0
Pelayo, J et al. [47]	2	2	1	2	0	2	2	1
Alkundi, A et al.[48]	2	2	1	2	0	2	2	1
Qin, C et al. [49]	2	2	1	2	0	2	2	2
Chen, Y et al.[50]	2	2	1	2	0	2	2	2
Khamis, F et al. [51]	2	2	2	2	0	2	2	0
Gayam, V et al.[52]	2	2	1	2	0	2	2	1
Shi, Q et al.[53]	2	2	1	2	0	2	2	1
Nowak, B et al.[54]	2	2	1	2	0	2	2	1
Rottoli, M et al.[55]	2	2	1	2	0	2	2	1
Huang, Y et al.[56]	1	2	1	1	0	2	2	0
Li, T et al. [57]	2	2	1	2	0	2	2	1
Zhou, X et al.[58]	2	2	1	2	0	2	2	1
Wu, F et al.[59]	1	2	1	2	0	2	2	2
Xu, J et al.[60]	2	2	1	2	0	2	2	1
Buckner, FS et al.[61]	2	2	1	2	0	2	2	1
Wan, S et al.[62]	2	2	2	2	0	2	2	1
Li, J et al.[63]	2	2	1	1	0	2	2	0
Cao, M et al.[64]	2	2	1	2	0	2	2	1
Sun, L et al. [65]	2	2	1	2	0	2	2	0
Huang, R et al.[66]	2	2	1	2	0	2	2	1
Labenz, C et al.[67]	2	2	1	2	0	2	2	0
Zhang, JJ et al. [68]	2	2	2	2	0	2	2	1
Pérez, FM et al.[69]	2	2	0	2	0	2	2	0
Wang, L et al.[70]	2	2	2	2	0	2	2	1
Wang, D et al.[71]	2	2	1	2	0	2	2	1
Güner, R et al. [72]	2	2	1	2	0	2	2	1

Zhou, Y et al.[78]	2	2	1	2	0	2	2	1
Feng, Y et al.[74]	2	2	1	2	0	2	2	1
Brill, SE et al.[75]	2	2	1	2	0	2	2	1
Shah, P et al.[81]	2	2	1	2	0	2	2	2
Lagi, F et al.[77]	2	2	1	2	0	2	2	1
Yang, Q et al.[78]	2	2	1	2	0	2	2	1
Ji, W et al.[79]	2	2	1	2	0	2	2	2
Lee, JY et al.[80]	2	2	1	2	0	2	2	2
Shahriarirad, R et al. [81]	2	2	1	2	0	2	2	1
Baqui, P et al.[82]	2	2	0	2	0	2	2	2
Asfahan, S et al.[83]	2	1	1	2	0	2	2	2
Petrilli, CM et al.[84]	2	2	2	2	0	2	2	2
Mendy, A et al.[85]	2	2	0	2	0	2	2	2
Masetti, C et al. [86]	2	2	1	2	0	2	2	1
Davies, MA et al. [87]	2	2	1	2	0	2	2	2
Huang, J et al.[88]	2	2	1	2	0	2	2	1
Kammar-Garcia, A et al. [89]	2	2	0	2	0	2	2	2
Ye, C et al.[90]	2	2	1	2	0	2	2	2
Bonetti, G et al.[91]	2	2	1	2	0	2	2	1
Ponziani, FR et al.[92]	2	2	1	2	0	2	2	2
Klang, E et al.[93]	2	2	1	2	0	2	2	2
Sousa, GJB et al.[94]	2	2	1	2	0	2	2	2
Brouns, SH et al.[95]	2	2	0	2	0	2	2	1
Lorente-Ros, A et al.[96]	2	2	2	2	0	2	2	2
Tatum, D et al.[97]	2	2	2	2	0	2	2	1
Sabri, A et al.[98]	2	2	1	2	0	2	2	0
Cai, Q et al.[99]	2	2	1	2	0	2	2	1
Pettit, NN et al.[100]	2	2	1	2	0	2	2	1
Ji, D et al.[101]	2	2	1	2	0	2	2	1

Gidari, A et al.[102]	2	2	1	2	0	2	2	0
Du, RH et al.[103]	2	2	2	2	0	2	2	1
Sarin, SK et al. [104]	2	2	1	2	0	2	2	1
Ebinger, JE et al.[105]	2	2	1	2	0	2	2	1
Pagnesi, M et al.[106]	2	2	2	2	0	2	2	2
Rastad, H et al.[107]	2	2	1	2	0	2	2	2
van Gerwen, M et al. [108]	2	2	1	2	0	2	2	2
Zhou, Y et al.[109]	2	2	1	2	0	2	2	0
Grasselli, G et al.[110]	2	2	1	2	0	2	2	2
Xu, PP et al.[111]	2	2	1	2	0	2	2	2
Cen, Y et al.[112]	2	2	2	2	0	2	2	2
Soares, RCM et al.[113]	2	2	0	2	0	0	2	2
Li, X et al.[114]	2	2	2	2	0	2	2	2
Gao, S et al. [115]	2	2	1	2	0	2	2	1
Urra, JM et al.[116]	2	2	1	2	0	2	2	1
Rivera-Izquierdo, M et al.[117]	2	2	1	2	0	2	2	1
Deng, Q et al.[118]	2	2	1	2	0	2	2	1
Kim, DW et al.[119]	2	2	1	2	0	2	2	2
Hewitt, J et al.[120]	2	2	2	2	0	2	2	2

Supplementary Table 3

Assessment of publication bias and small study effect for primary outcomes

	**Comorbidities**		**Severity**		**Mortality**	
	**Beta (95% CI)**	**p-value**	**Beta (95% CI)**	**p-value**	**Beta (95% CI)**	**p-value**
One comorbidity	1·83 (-0·30, 3·97)	0·09	0·48 (-0·55, 1·51)	0·35	1·07 (-1·06, 3·20)	0·32
No comorbidity	-1·87 (-4·07, 0·33)	0·09	0·22 (-0·54, 0·97)	0·56	1·17 (-0·04, 2·39)	0·06
Multiple comorbidities	0·29 (-8·61, 9·20)	0·94	0·06 (-4·88, 5·00)	0·98	-1·36 (-3·61, 0·88)	0·18
HTN	1·62 (0·06, 3·17)	0·041	0·78 (0·19, 1·37)	0·011	0·65 (-0·85, 2·14)	0·39
DM	0·71 (-0·39, 1·81)	0·20	0·80 (0·34, 1·27)	0·001	-0·16 (-1·4, 1·09)	0·80
CVD	0·19 (-1·27, 1·64)	0·8	1·00 (0·53, 1·46)	< 0·001	-0·19 (-1·28, 0·9)	0·73
Obesity	4·68 (0·34, 9·02)	0·036	0·71 (-1·24, 2·66)	0·45	0·47 (-2·31, 3·25)	0·71
CVA	-0·13 (-0·52, 0·26)	0·50	0·69 (0·18, 1·20)	0·011	0·75 (-0·16, 1·65)	0·10
Lung disease	0·22 (-0·54, 0·99)	0·56	0·85 (0·47, 1·23)	< 0·001	0·52 (-0·33, 1·36)	0·22
Cancer/malignancy	0·48 (-0·18, 1·13)	0·15	0·42 (-0·07, 0·90)	0·09	-0·12 (-0·82, 0·58)	0·73
CKD/other renal disease	0·38 (-0·72, 1·48)	0·49	0·51 (-0·24, 1·26)	0·17	0·28 (-0·83, 1·40)	0·61
Liver disease	0·30 (-0·77, 1·37)	0·57	0·34 (0·04, 0·64)	0·026	0·67 (-0·43, 1·77)	0·22

Beta regression coefficient, *CI* confidence interval, *HTN* Hypertension, *DM* Diabetes mellitus, *CVD* Cardiovascular disease, *CVA* Cerebrovascular accident, *CKD* Chronic kidney disease.

Supplementary Table 4

Demographic characteristics by geographic regions

**Demographic characteristics**	**Overall**	**Asia**	**Europe**	**Latin America**	**USA**	**South Africa**
Number of studies	120	72	26	5	16	1
Number of subjects (%)	125446	63462<br>(50·6%)	13929<br>(11·1%)	24575<br>(19·6%)	20602<br>(16·4%)	2978<br>(2·4%)
Age; mean<br>(95% CI)	56·2<br>(52·6, 59·8)	54·4<br>(50·8, 58·0)	64·8<br>(61·9, 67·8)	50·2<br>(42·6, 57·9)	60·4<br>(57·7, 63·1)	NA
Gender[$]<br>Total<br>Female<br>Meta-proportion of female;<br>(95% CI)	 104670<br>48446 (46·3)<br>0·45<br>(0·43, 0·47)	 42587<br>22056 (51·8)<br>0·47<br>(0·45, 0·50)	 13929<br>5386 (38·7)<br>0·39<br>(0·34, 0·45)	 24575<br>10536 (42·9)<br>0·44<br>(0·41, 0·48)	 20601<br>8620 (41·8)<br>0·45<br>(0·42, 0·47)	 2978<br>1848 (62·0)<br>0·62<br>(0·60, 0·64)

NA Not available, *CI* confidence interval.
[$]Gender information was not available for 20875 patients from Asian study and 1 patient from a USA study, meta-proportion for female gender was calculated after excluding a European study that included only pregnant women.

Supplementary Table 5

Comparison of proportion estimates of underlying medical diseases and associated COVID-19 severity and mortality between geographic regions

	**Asia vs. Europe**	**Asia vs. Latin America**	**Asia vs. USA**	**Europe vs. Latin America**	**Europe vs. USA**	**Latin America vs. USA**
	**p-value**	**p-value**	**p-value**	**p-value**	**p-value**	**p-value**
**Comorbidities**						

One comorbidity	<0.001	0.768	<0.001	0.055	0.009	0.001
Multiple comorbidities	0.273	0.837	0.056	0.309	0.677	0.124
HTN	<0.001	0.659	<001	0.583	0.045	0.235
DM	0.010	0.239	<0.001	0.761	<0.001	0.006
CVD	<0.001	0.027	<0.001	0.846	0.113	0.221
Obesity	0.257	0.640	0.640	0.287	0.006	0.001
CVA	0.852	NA	0.119	0.657	0.852	0.657
Lung disease	0.002	0.936	<0.001	0.047	0.036	0.001
Cancer/malignancy	<0.001	NA	<0.001	NA	0.417	NA
CKD/other renal disease	0.003	0.814	<0.001	0.067	0.064	0.001
Liver disease	0.673	0.601	0.580	0.950	0.972	0.911
**Severity**						
Total	0.526	0.897	0.046	0.629	0.052	0.328
One comorbidity	0.021	0.914	0.296	0.209	0.235	0.649
No comorbidity	0.250	0.611	0.463	0.640	0.598	0.985
Multiple comorbidities	0.049	0.519	0.027	0.017	0.579	0.002
HTN	0.027	0.251	0.071	0.710	0.574	0.563
DM	0.037	0.711	0.022	0.761	0.914	0.787
CVD	0.158	0.447	0.033	0.615	0.522	0.711

Obesity	0.447	0.816	0.582	0.730	0.665	0.878
CVA	0.531	NA	0.026	0.713	0.531	0.713
Lung disease	0.216	0.661	0.035	0.830	0.590	0.905
Cancer/malignancy	0.535	NA	0.715	NA	0.751	0.751
CKD/other renal disease	0.027	NA	<0.001	NA	0.583	0.583
Liver disease	0.660	0.660	0.442	1.00	0.824	0.824
**Mortality**						
Total	0.045	0.436	0.427	0.546	0.450	0.943
One comorbidity	0.096	0.558	0.866	0.576	0.293	0.721
No comorbidity	0.112	0.221	0.943	0.957	0.313	0.389
Multiple comorbidities	0.139	NA	0.065	0.273	NA	0.273
HTN	0.120	NA	0.888	NA	0.245	0.245
DM	0.001	<0.001	0.340	0.131	0.025	0.001
CVD	0.033	0.011	0.145	0.299	0.616	0.170
Obesity	0.072	NA	0.003	0.072	0.160	0.003
CVA	NA	NA	0.672	NA	NA	0.672
Lung disease	0.218	0.060	0.465	0.360	0.075	0.023
Cancer/malignancy	0.035	NA	0.508	0.096	0.035	0.096
CKD/other renal disease	0.011	<0.001	0.499	0.118	0.020	0.001

Liver disease	0.166	0.034	0.829	0.500	0.284	0.080

NA Not available, *HTN* Hypertension, *DM* Diabetes mellitus, *CVD* Cardiovascular disease, *CVA* Cerebrovascular accident, *CKD* Chronic kidney disease.

Supplementary Table 6

Comparison of proportion estimates of underlying medical diseases and associated COVID-19 severity and mortality between age groups

	**Age ≤ 50 years Vs 50 years < Age ≤ 65 years**	**Age ≤ 50 years Vs Age > 65 years**	**50 years < Age ≤ 65 years vs. Age > 65 years**
	**p-value**	**p-value**	**p-value**
**Comorbidities**			
One comorbidity	<0.001	<0.001	0.001
Multiple comorbidities	0.001	0.022	0.278
HTN	<0.001	<0.001	0.001
DM	0.023	<0.001	0.008
CVD	0.666	<0.001	<0.001
Obesity	0.094	0.286	0.562
CVA	0.405	0.157	0.070
Lung disease	0.257	0.018	0.076
Cancer/malignancy	0.047	0.004	0.068
CKD/other renal disease	0.273	<0.001	<0.001
Liver disease	0.474	0.935	0.542
**Severity**			

One comorbidity	0.886	0.866	0.790
No comorbidity	0.127	0.482	0.884
Multiple comorbidities	0.001	NA	NA
HTN	0.981	0.646	0.610
DM	0.850	0.511	0.557
CVD	0.423	0.764	0.712
Obesity	0.991	0.508	0.471
CVA	0.442	0.893	0.667
Lung disease	0.362	0.631	0.927
Cancer/malignancy	0.486	0.615	0.331
CKD/other renal disease	0.122	0.161	0.799
Liver disease	0.977	0.629	0.625
**Mortality**			
One comorbidity	0.153	0.010	0.064
No comorbidity	0.110	0.054	0.311
Multiple comorbidities	0.205	0.558	0.885
HTN	0.635	0.447	0.610
DM	0.794	0.823	0.479
CVD	0.580	0.246	0.297

Obesity	0.615	0.743	0.442
CVA	0.416	0.199	0.296
Lung disease	0.744	0.716	0.187
Cancer/malignancy	0.319	0.254	0.651
CKD/other renal disease	0.534	0.319	0.447
Liver disease	0.318	0.503	0.957

NA Not available, *HTN* Hypertension, *DM* Diabetes mellitus, *CVD* Cardiovascular disease, *CVA* Cerebrovascular accident, *CKD* Chronic kidney disease.

References

1 Wang, L. *et al.* Coronavirus disease 2019 in elderly patients: Characteristics and prognostic factors based on 4-week follow-up. *J Infect* **80**, 639-645, doi:10.1016/j.jinf.2020.03.019 (2020).

2 Cao, J. *et al.* Clinical Features and Short-term Outcomes of 102 Patients with Coronavirus Disease 2019 in Wuhan, China. *Clin Infect Dis* **71**, 748-755, doi:10.1093/cid/ciaa243 (2020).

3 Chen, T. *et al.* Clinical characteristics of 113 deceased patients with coronavirus disease 2019: retrospective study. *BMJ* **368**, m1091, doi:10.1136/bmj.m1091 (2020).

4 Deng, Y. *et al.* Clinical characteristics of fatal and recovered cases of coronavirus disease 2019 in Wuhan, China: a retrospective study. *Chin Med J (Engl)* **133**, 1261-1267, doi:10.1097/CM9.0000000000000824 (2020).

5 Yuan, M., Yin, W., Tao, Z., Tan, W. & Hu, Y. Association of radiologic findings with mortality of patients infected with 2019 novel coronavirus in Wuhan, China. *PLoS One* **15**, e0230548, doi:10.1371/journal.pone.0230548 (2020).

6 Wu, C. *et al.* Risk Factors Associated With Acute Respiratory Distress Syndrome and Death in Patients With Coronavirus Disease 2019 Pneumonia in Wuhan, China. *JAMA Intern Med* **180**, 934-943, doi:10.1001/jamainternmed.2020.0994 (2020).

7 Li, K. *et al.* The Clinical and Chest CT Features Associated With Severe and Critical COVID-19 Pneumonia. *Invest Radiol* **55**, 327-331, doi:10.1097/RLI.0000000000000672 (2020).

8 Yang, X. *et al.* Clinical course and outcomes of critically ill patients with SARS-CoV-2 pneumonia in Wuhan, China: a single-centered, retrospective, observational study. *Lancet Respir Med* **8**, 475-481, doi:10.1016/S2213-2600(20)30079-5 (2020).

9 Zhang, J. J. *et al.* Clinical characteristics of 140 patients infected with SARS-CoV-2 in Wuhan, China. *Allergy* **75**, 1730-1741, doi:10.1111/all.14238 (2020).

10 Wang, D. *et al.* Clinical Characteristics of 138 Hospitalized Patients With 2019 Novel Coronavirus-Infected Pneumonia in Wuhan, China. *JAMA* **323**, 1061-1069, doi:10.1001/jama.2020.1585 (2020).

11 Huang, C. *et al.* Clinical features of patients infected with 2019 novel coronavirus in Wuhan, China. *Lancet* **395**, 497-506, doi:10.1016/S0140-6736(20)30183-5 (2020).

12 Wang, Z., Yang, B., Li, Q., Wen, L. & Zhang, R. Clinical Features of 69 Cases With Coronavirus Disease 2019 in Wuhan, China. *Clin Infect Dis* **71**, 769-777, doi:10.1093/cid/ciaa272 (2020).

13 Guan, W. J. *et al.* Comorbidity and its impact on 1590 patients with COVID-19 in China: a nationwide analysis. *Eur Respir J* **55**, doi:10.1183/13993003.00547-2020 (2020).

14 Fu, L. *et al.* Clinical characteristics of coronavirus disease 2019 (COVID-19) in China: A systematic review and meta-analysis. *J Infect* **80**, 656-665, doi:10.1016/j.jinf.2020.03.041 (2020).

15 Cai, Q. *et al.* COVID-19 in a designated infectious diseases hospital outside Hubei Province, China. *Allergy* **75**, 1742-1752, doi:10.1111/all.14309 (2020).

16 Zhang, G. *et al.* Clinical features and short-term outcomes of 221 patients with COVID-19 in Wuhan, China. *J Clin Virol* **127**, 104364, doi:10.1016/j.jcv.2020.104364 (2020).

17 Zhang, R. *et al.* CT features of SARS-CoV-2 pneumonia according to clinical presentation: a retrospective analysis of 120 consecutive patients from Wuhan city. *Eur Radiol* **30**, 4417-4426, doi:10.1007/s00330-020-06854-1 (2020).

18 Nikpouraghdam, M. *et al.* Epidemiological characteristics of coronavirus disease 2019 (COVID-19) patients in IRAN: A single center study. *J Clin Virol* **127**, 104378, doi:10.1016/j.jcv.2020.104378 (2020).

19 Li, Y., Hu, Y., Yu, J. & Ma, T. Retrospective analysis of laboratory testing in 54 patients with severe- or critical-type 2019 novel coronavirus pneumonia. *Lab Invest* **100**, 794-800, doi:10.1038/s41374-020-0431-6 (2020).

20 Zhou, F. *et al.* Clinical course and risk factors for mortality of adult inpatients with COVID-19 in Wuhan, China: a retrospective cohort study. *Lancet* **395**, 1054-1062, doi:10.1016/S0140-6736(20)30566-3 (2020).

21 He, R. *et al.* The clinical course and its correlated immune status in COVID-19 pneumonia. *J Clin Virol* **127**, 104361, doi:10.1016/j.jcv.2020.104361 (2020).

22 Yang, A. P., Liu, J. P., Tao, W. Q. & Li, H. M. The diagnostic and predictive role of NLR, d-NLR and PLR in COVID-19 patients. *Int Immunopharmacol* **84**, 106504, doi:10.1016/j.intimp.2020.106504 (2020).

23 Borobia, A. M. *et al.* A Cohort of Patients with COVID-19 in a Major Teaching Hospital in Europe. *J Clin Med* **9**, doi:10.3390/jcm9061733 (2020).

24 Zhang, C. *et al.* A Novel Scoring System for Prediction of Disease Severity in COVID-19. *Front Cell Infect Microbiol* **10**, 318, doi:10.3389/fcimb.2020.00318 (2020).

25 Zhao, J., Gao, H. Y., Feng, Z. Y. & Wu, Q. J. A Retrospective Analysis of the Clinical and Epidemiological Characteristics of COVID-19 Patients in Henan Provincial People's Hospital, Zhengzhou, China. *Front Med (Lausanne)* **7**, 286, doi:10.3389/fmed.2020.00286 (2020).

26 Yao, Q. *et al.* A retrospective study of risk factors for severe acute respiratory syndrome coronavirus 2 infections in hospitalized adult patients. *Pol Arch Intern Med* **130**, 390-399, doi:10.20452/pamw.15312 (2020).

27 Kayem, G. *et al.* A snapshot of the Covid-19 pandemic among pregnant women in France. *J Gynecol Obstet Hum Reprod* **49**, 101826, doi:10.1016/j.jogoh.2020.101826 (2020).

28 Tambe, M. P. *et al.* An epidemiological study of laboratory confirmed COVID-19 cases admitted in a tertiary care hospital of Pune, Maharashtra. *Indian J Public Health* **64**, S183-S187, doi:10.4103/ijph.IJPH_522_20 (2020).

29 Yang, Q. *et al.* Analysis of the clinical characteristics, drug treatments and prognoses of 136 patients with coronavirus disease 2019. *J Clin Pharm Ther* **45**, 609-616, doi:10.1111/jcpt.13170 (2020).

30 Senkal, N. *et al.* Association between chronic ACE inhibitor exposure and decreased odds of severe disease in patients with COVID-19. *Anatol J Cardiol* **24**, 21-29, doi:10.14744/AnatolJCardiol.2020.57431 (2020).

31 Shi, S. *et al.* Association of Cardiac Injury With Mortality in Hospitalized Patients With COVID-19 in Wuhan, China. *JAMA Cardiol* **5**, 802-810, doi:10.1001/jamacardio.2020.0950 (2020).

32 Mani, V. R., Kalabin, A., Valdivieso, S. C., Murray-Ramcharan, M. & Donaldson, B. New York Inner City Hospital COVID-19 Experience and Current Data: Retrospective Analysis at the Epicenter of the American Coronavirus Outbreak. *J Med Internet Res* **22**, e20548, doi:10.2196/20548 (2020).

33 Li, M. *et al.* Cardiovascular disease potentially contributes to the progression and poor prognosis of COVID-19. *Nutr Metab Cardiovasc Dis* **30**, 1061-1067, doi:10.1016/j.numecd.2020.04.013 (2020).
34 Salacup, G. *et al.* Characteristics and clinical outcomes of COVID-19 patients in an underserved-inner city population: A single tertiary center cohort. *J Med Virol*, doi:10.1002/jmv.26252 (2020).
35 Shi, S. *et al.* Characteristics and clinical significance of myocardial injury in patients with severe coronavirus disease 2019. *Eur Heart J* **41**, 2070-2079, doi:10.1093/eurheartj/ehaa408 (2020).
36 Kalyanaraman Marcello, R. *et al.* Characteristics and Outcomes of COVID-19 Patients in New York City's Public Hospital System. *medRxiv*, doi:10.1101/2020.05.29.20086645 (2020).
37 Israelsen, S. B. *et al.* Characteristics of patients with COVID-19 pneumonia at Hvidovre Hospital, March-April 2020. *Dan Med J* **67** (2020).
38 Pellaud, C. *et al.* Characteristics, comorbidities, 30-day outcome and in-hospital mortality of patients hospitalised with COVID-19 in a Swiss area - a retrospective cohort study. *Swiss Med Wkly* **150**, w20314, doi:10.4414/smw.2020.20314 (2020).
39 Gregoriano, C. *et al.* Characteristics, predictors and outcomes among 99 patients hospitalised with COVID-19 in a tertiary care centre in Switzerland: an observational analysis. *Swiss Med Wkly* **150**, w20316, doi:10.4414/smw.2020.20316 (2020).
40 Argenziano, M. G. *et al.* Characterization and clinical course of 1000 Patients with COVID-19 in New York: retrospective case series. *medRxiv*, doi:10.1101/2020.04.20.20072116 (2020).
41 Borghesi, A. *et al.* Chest X-ray severity index as a predictor of in-hospital mortality in coronavirus disease 2019: A study of 302 patients from Italy. *Int J Infect Dis* **96**, 291-293, doi:10.1016/j.ijid.2020.05.021 (2020).
42 Toussie, D. *et al.* Clinical and Chest Radiography Features Determine Patient Outcomes in Young and Middle-aged Adults with COVID-19. *Radiology* **297**, E197-E206, doi:10.1148/radiol.2020201754 (2020).
43 Ortiz-Brizuela, E. *et al.* Clinical and Epidemiological Characteristics of Patients Diagnosed with Covid-19 in a Tertiary Care Center in Mexico City: A Prospective Cohort Study. *Rev Invest Clin* **72**, 165-177, doi:10.24875/RIC.20000211 (2020).
44 Aggarwal, A., Shrivastava, A., Kumar, A. & Ali, A. Clinical and Epidemiological Features of SARS-CoV-2 Patients in SARI Ward of a Tertiary Care Centre in New Delhi. *J Assoc Physicians India* **68**, 19-26 (2020).
45 Javanian, M. *et al.* Clinical and laboratory findings from patients with COVID-19 pneumonia in Babol North of Iran: a retrospective cohort study. *Rom J Intern Med* **58**, 161-167, doi:10.2478/rjim-2020-0013 (2020).
46 Huang, Q. *et al.* Clinical characteristics and drug therapies in patients with the common-type coronavirus disease 2019 in Hunan, China. *Int J Clin Pharm* **42**, 837-845, doi:10.1007/s11096-020-01031-2 (2020).
47 Pelayo, J. *et al.* Clinical Characteristics and Outcomes of Community- and Hospital-Acquired Acute Kidney Injury with COVID-19 in a US Inner City Hospital System. *Cardiorenal Med* **10**, 223-231, doi:10.1159/000509182 (2020).
48 Alkundi, A., Mahmoud, I., Musa, A., Naveed, S. & Alshawwaf, M. Clinical characteristics and outcomes of COVID-19 hospitalized patients with diabetes in the United Kingdom: A retrospective single centre study. *Diabetes Res Clin Pract* **165**, 108263, doi:10.1016/j.diabres.2020.108263 (2020).
49 Qin, C. *et al.* Clinical Characteristics and Outcomes of COVID-19 Patients With a History of Stroke in Wuhan, China. *Stroke* **51**, 2219-2223, doi:10.1161/STROKEAHA.120.030365 (2020).

50 Chen, Y. *et al.* Clinical Characteristics and Outcomes of Patients With Diabetes and COVID-19 in Association With Glucose-Lowering Medication. *Diabetes Care* **43**, 1399-1407, doi:10.2337/dc20-0660 (2020).
51 Khamis, F. *et al.* Clinical characteristics and outcomes of the first 63 adult patients hospitalized with COVID-19: An experience from Oman. *J Infect Public Health* **13**, 906-913, doi:10.1016/j.jiph.2020.06.002 (2020).
52 Gayam, V. *et al.* Clinical characteristics and predictors of mortality in African-Americans with COVID-19 from an inner-city community teaching hospital in New York. *J Med Virol*, doi:10.1002/jmv.26306 (2020).
53 Shi, Q. *et al.* Clinical Characteristics and Risk Factors for Mortality of COVID-19 Patients With Diabetes in Wuhan, China: A Two-Center, Retrospective Study. *Diabetes Care* **43**, 1382-1391, doi:10.2337/dc20-0598 (2020).
54 Nowak, B. *et al.* Clinical characteristics and short-term outcomes of patients with coronavirus disease 2019: a retrospective single-center experience of a designated hospital in Poland. *Pol Arch Intern Med* **130**, 407-411, doi:10.20452/pamw.15361 (2020).
55 Rottoli, M. *et al.* How important is obesity as a risk factor for respiratory failure, intensive care admission and death in hospitalised COVID-19 patients? Results from a single Italian centre. *Eur J Endocrinol* **183**, 389-397, doi:10.1530/EJE-20-0541 (2020).
56 Huang, Y. *et al.* Clinical characteristics of 17 patients with COVID-19 and systemic autoimmune diseases: a retrospective study. *Ann Rheum Dis* **79**, 1163-1169, doi:10.1136/annrheumdis-2020-217425 (2020).
57 Li, T. *et al.* Clinical characteristics of 312 hospitalized older patients with COVID-19 in Wuhan, China. *Arch Gerontol Geriatr* **91**, 104185, doi:10.1016/j.archger.2020.104185 (2020).
58 Zhou, X., Zhu, J. & Xu, T. Clinical characteristics of coronavirus disease 2019 (COVID-19) patients with hypertension on renin-angiotensin system inhibitors. *Clin Exp Hypertens* **42**, 656-660, doi:10.1080/10641963.2020.1764018 (2020).
59 Wu, F. *et al.* Clinical characteristics of COVID-19 infection in chronic obstructive pulmonary disease: a multicenter, retrospective, observational study. *J Thorac Dis* **12**, 1811-1823, doi:10.21037/jtd-20-1914 (2020).
60 Xu, J. *et al.* Clinical course and predictors of 60-day mortality in 239 critically ill patients with COVID-19: a multicenter retrospective study from Wuhan, China. *Crit Care* **24**, 394, doi:10.1186/s13054-020-03098-9 (2020).
61 Buckner, F. S. *et al.* Clinical Features and Outcomes of 105 Hospitalized patients with COVID-19 in Seattle, Washington. *Clin Infect Dis*, doi:10.1093/cid/ciaa632 (2020).
62 Wan, S. *et al.* Clinical features and treatment of COVID-19 patients in northeast Chongqing. *J Med Virol* **92**, 797-806, doi:10.1002/jmv.25783 (2020).
63 Li, J. *et al.* Clinical features of familial clustering in patients infected with 2019 novel coronavirus in Wuhan, China. *Virus Res* **286**, 198043, doi:10.1016/j.virusres.2020.198043 (2020).
64 Cao, M. *et al.* Clinical Features of Patients Infected with the 2019 Novel Coronavirus (COVID-19) in Shanghai, China. *medRxiv*, doi:10.1101/2020.03.04.20030395 (2020).
65 Sun, L. *et al.* Clinical features of patients with coronavirus disease 2019 from a designated hospital in Beijing, China. *J Med Virol*, doi:10.1002/jmv.25966 (2020).
66 Huang, R. *et al.* Clinical findings of patients with coronavirus disease 2019 in Jiangsu province, China: A retrospective, multi-center study. *PLoS Negl Trop Dis* **14**, e0008280, doi:10.1371/journal.pntd.0008280 (2020).

67 Labenz, C. *et al.* Clinical Frailty Scale for risk stratification in patients with SARS-CoV-2 infection. *J Investig Med* **68**, 1199-1202, doi:10.1136/jim-2020-001410 (2020).
68 Zhang, J. J. *et al.* Clinical, radiological, and laboratory characteristics and risk factors for severity and mortality of 289 hospitalized COVID-19 patients. *Allergy*, doi:10.1111/all.14496 (2020).
69 Martos Perez, F. *et al.* Comorbidity and prognostic factors on admission in a COVID-19 cohort of a general hospital. *Rev Clin Esp*, doi:10.1016/j.rce.2020.05.017 (2020).
70 Wang, L. *et al.* Coronavirus Disease 19 Infection Does Not Result in Acute Kidney Injury: An Analysis of 116 Hospitalized Patients from Wuhan, China. *Am J Nephrol* **51**, 343-348, doi:10.1159/000507471 (2020).
71 Cai, Q. *et al.* Obesity and COVID-19 Severity in a Designated Hospital in Shenzhen, China. *Diabetes Care* **43**, 1392-1398, doi:10.2337/dc20-0576 (2020).
72 Guner, R. *et al.* COVID-19 experience of the major pandemic response center in the capital: Results of the pandemic's first month in Turkey. *Turk J Med Sci*, doi:10.3906/sag-2006-164 (2020).
73 Zhou, Y. *et al.* COVID-19 Is Distinct From SARS-CoV-2-Negative Community-Acquired Pneumonia. *Front Cell Infect Microbiol* **10**, 322, doi:10.3389/fcimb.2020.00322 (2020).
74 Feng, Y. *et al.* COVID-19 with Different Severities: A Multicenter Study of Clinical Features. *Am J Respir Crit Care Med* **201**, 1380-1388, doi:10.1164/rccm.202002-0445OC (2020).
75 Brill, S. E. *et al.* COVID-19: a retrospective cohort study with focus on the over-80s and hospital-onset disease. *BMC Med* **18**, 194, doi:10.1186/s12916-020-01665-z (2020).
76 Shah, P. *et al.* Demographics, comorbidities and outcomes in hospitalized Covid-19 patients in rural southwest Georgia. *Ann Med* **52**, 354-360, doi:10.1080/07853890.2020.1791356 (2020).
77 Lagi, F. *et al.* Early experience of an infectious and tropical diseases unit during the coronavirus disease (COVID-19) pandemic, Florence, Italy, February to March 2020. *Euro Surveill* **25**, doi:10.2807/1560-7917.ES.2020.25.17.2000556 (2020).
78 Yang, Q. *et al.* Effect of hypertension on outcomes of adult inpatients with COVID-19 in Wuhan, China: a propensity score-matching analysis. *Respir Res* **21**, 172, doi:10.1186/s12931-020-01435-8 (2020).
79 Ji, W. *et al.* Effect of Underlying Comorbidities on the Infection and Severity of COVID-19 in Korea: a Nationwide Case-Control Study. *J Korean Med Sci* **35**, e237, doi:10.3346/jkms.2020.35.e237 (2020).
80 Lee, J. Y. *et al.* Epidemiological and clinical characteristics of coronavirus disease 2019 in Daegu, South Korea. *Int J Infect Dis* **98**, 462-466, doi:10.1016/j.ijid.2020.07.017 (2020).
81 Shahriarirad, R. *et al.* Epidemiological and clinical features of 2019 novel coronavirus diseases (COVID-19) in the South of Iran. *BMC Infect Dis* **20**, 427, doi:10.1186/s12879-020-05128-x (2020).
82 Baqui, P., Bica, I., Marra, V., Ercole, A. & van der Schaar, M. Ethnic and regional variations in hospital mortality from COVID-19 in Brazil: a cross-sectional observational study. *Lancet Glob Health* **8**, e1018-e1026, doi:10.1016/S2214-109X(20)30285-0 (2020).
83 Asfahan, S. *et al.* Extrapolation of mortality in COVID-19: Exploring the role of age, sex, co-morbidities and health-care related occupation. *Monaldi Arch Chest Dis* **90**, doi:10.4081/monaldi.2020.1325 (2020).

84 Petrilli, C. M. *et al.* Factors associated with hospital admission and critical illness among 5279 people with coronavirus disease 2019 in New York City: prospective cohort study. *BMJ* **369**, m1966, doi:10.1136/bmj.m1966 (2020).
85 Mendy, A., Apewokin, S., Wells, A. A. & Morrow, A. L. Factors Associated with Hospitalization and Disease Severity in a Racially and Ethnically Diverse Population of COVID-19 Patients. *medRxiv*, doi:10.1101/2020.06.25.20137323 (2020).
86 Masetti, C. *et al.* High mortality in COVID-19 patients with mild respiratory disease. *Eur J Clin Invest* **50**, e13314, doi:10.1111/eci.13314 (2020).
87 Davies, M. A. HIV and risk of COVID-19 death: a population cohort study from the Western Cape Province, South Africa. *medRxiv*, doi:10.1101/2020.07.02.20145185 (2020).
88 Huang, J. *et al.* Hypoalbuminemia predicts the outcome of COVID-19 independent of age and co-morbidity. *J Med Virol*, doi:10.1002/jmv.26003 (2020).
89 Kammar-Garcia, A. *et al.* Impact of Comorbidities in Mexican Sars-Cov-2-Positive Patients: A Retrospective Analysis in a National Cohort. *Rev Invest Clin* **72**, 151-158, doi:10.24875/RIC.20000207 (2020).
90 Ye, C. *et al.* Impact of comorbidities on patients with COVID-19: A large retrospective study in Zhejiang, China. *J Med Virol*, doi:10.1002/jmv.26183 (2020).
91 Bonetti, G. *et al.* Laboratory predictors of death from coronavirus disease 2019 (COVID-19) in the area of Valcamonica, Italy. *Clin Chem Lab Med* **58**, 1100-1105, doi:10.1515/cclm-2020-0459 (2020).
92 Ponziani, F. R. *et al.* Liver involvement is not associated with mortality: results from a large cohort of SARS-CoV-2 positive patients. *Aliment Pharmacol Ther*, doi:10.1111/apt.15996 (2020).
93 Klang, E. *et al.* Severe Obesity as an Independent Risk Factor for COVID-19 Mortality in Hospitalized Patients Younger than 50. *Obesity (Silver Spring)* **28**, 1595-1599, doi:10.1002/oby.22913 (2020).
94 Sousa, G. J. B. *et al.* Mortality and survival of COVID-19. *Epidemiol Infect* **148**, e123, doi:10.1017/S0950268820001405 (2020).
95 Brouns, S. H. *et al.* Mortality and the Use of Antithrombotic Therapies Among Nursing Home Residents with COVID-19. *J Am Geriatr Soc* **68**, 1647-1652, doi:10.1111/jgs.16664 (2020).
96 Lorente-Ros, A. *et al.* Myocardial injury determination improves risk stratification and predicts mortality in COVID-19 patients. *Cardiol J*, doi:10.5603/CJ.a2020.0089 (2020).
97 Tatum, D. *et al.* Neutrophil-to-Lymphocyte Ratio and Outcomes in Louisiana Covid-19 Patients. *Shock*, doi:10.1097/SHK.0000000000001585 (2020).
98 Sabri, A. *et al.* Novel coronavirus disease 2019: predicting prognosis with a computed tomography-based disease severity score and clinical laboratory data. *Pol Arch Intern Med* **130**, 629-634, doi:10.20452/pamw.15422 (2020).
99 Driggin, E. *et al.* Cardiovascular Considerations for Patients, Health Care Workers, and Health Systems During the COVID-19 Pandemic. *J Am Coll Cardiol* **75**, 2352-2371, doi:10.1016/j.jacc.2020.03.031 (2020).
100 Pettit, N. N. *et al.* Obesity is Associated with Increased Risk for Mortality Among Hospitalized Patients with COVID-19. *Obesity (Silver Spring)* **28**, 1806-1810, doi:10.1002/oby.22941 (2020).

101 Ji, D. *et al.* Prediction for Progression Risk in Patients With COVID-19 Pneumonia: The CALL Score. *Clin Infect Dis* **71**, 1393-1399, doi:10.1093/cid/ciaa414 (2020).
102 Gidari, A., De Socio, G. V., Sabbatini, S. & Francisci, D. Predictive value of National Early Warning Score 2 (NEWS2) for intensive care unit admission in patients with SARS-CoV-2 infection. *Infect Dis (Lond)* **52**, 698-704, doi:10.1080/23744235.2020.1784457 (2020).
103 Du, R. H. *et al.* Predictors of mortality for patients with COVID-19 pneumonia caused by SARS-CoV-2: a prospective cohort study. *Eur Respir J* **55**, doi:10.1183/13993003.00524-2020 (2020).
104 Sarin, S. K. *et al.* Pre-existing liver disease is associated with poor outcome in patients with SARS CoV2 infection; The APCOLIS Study (APASL COVID-19 Liver Injury Spectrum Study). *Hepatol Int*, doi:10.1007/s12072-020-10072-8 (2020).
105 Ebinger, J. E. *et al.* Pre-existing traits associated with Covid-19 illness severity. *PLoS One* **15**, e0236240, doi:10.1371/journal.pone.0236240 (2020).
106 Pagnesi, M. *et al.* Pulmonary hypertension and right ventricular involvement in hospitalised patients with COVID-19. *Heart* **106**, 1324-1331, doi:10.1136/heartjnl-2020-317355 (2020).
107 Rastad, H. *et al.* Risk and predictors of in-hospital mortality from COVID-19 in patients with diabetes and cardiovascular disease. *Diabetol Metab Syndr* **12**, 57, doi:10.1186/s13098-020-00565-9 (2020).
108 van Gerwen, M. *et al.* Risk factors and outcomes of COVID-19 in New York City; a retrospective cohort study. *J Med Virol*, doi:10.1002/jmv.26337 (2020).
109 Zhou, Y., Zhang, Z., Tian, J. & Xiong, S. Risk factors associated with disease progression in a cohort of patients infected with the 2019 novel coronavirus. *Ann Palliat Med* **9**, 428-436, doi:10.21037/apm.2020.03.26 (2020).
110 Grasselli, G. *et al.* Risk Factors Associated With Mortality Among Patients With COVID-19 in Intensive Care Units in Lombardy, Italy. *JAMA Intern Med*, doi:10.1001/jamainternmed.2020.3539 (2020).
111 Xu, P. P. *et al.* Risk factors for adverse clinical outcomes with COVID-19 in China: a multicenter, retrospective, observational study. *Theranostics* **10**, 6372-6383, doi:10.7150/thno.46833 (2020).
112 Cen, Y. *et al.* Risk factors for disease progression in patients with mild to moderate coronavirus disease 2019-a multi-centre observational study. *Clin Microbiol Infect*, doi:10.1016/j.cmi.2020.05.041 (2020).
113 Soares, R. C. M., Mattos, L. R. & Raposo, L. M. Risk Factors for Hospitalization and Mortality due to COVID-19 in Espirito Santo State, Brazil. *Am J Trop Med Hyg* **103**, 1184-1190, doi:10.4269/ajtmh.20-0483 (2020).
114 Li, X. *et al.* Risk factors for severity and mortality in adult COVID-19 inpatients in Wuhan. *J Allergy Clin Immunol* **146**, 110-118, doi:10.1016/j.jaci.2020.04.006 (2020).
115 Gao, S. *et al.* Risk factors influencing the prognosis of elderly patients infected with COVID-19: a clinical retrospective study in Wuhan, China. *Aging (Albany NY)* **12**, 12504-12516, doi:10.18632/aging.103631 (2020).
116 Urra, J. M., Cabrera, C. M., Porras, L. & Rodenas, I. Selective CD8 cell reduction by SARS-CoV-2 is associated with a worse prognosis and systemic inflammation in COVID-19 patients. *Clin Immunol* **217**, 108486, doi:10.1016/j.clim.2020.108486 (2020).
117 Rivera-Izquierdo, M. *et al.* Sociodemographic, clinical and laboratory factors on admission associated with COVID-19 mortality in hospitalized patients: A retrospective observational study. *PLoS One* **15**, e0235107, doi:10.1371/journal.pone.0235107 (2020).

118 Deng, Q. *et al.* Suspected myocardial injury in patients with COVID-19: Evidence from front-line clinical observation in Wuhan, China. *Int J Cardiol* **311**, 116-121, doi:10.1016/j.ijcard.2020.03.087 (2020).
119 Kim, D. W., Byeon, K. H., Kim, J., Cho, K. D. & Lee, N. The Correlation of Comorbidities on the Mortality in Patients with COVID-19: an Observational Study Based on the Korean National Health Insurance Big Data. *J Korean Med Sci* **35**, e243, doi:10.3346/jkms.2020.35.e243 (2020).
120 Hewitt, J. *et al.* The effect of frailty on survival in patients with COVID-19 (COPE): a multicentre, European, observational cohort study. *Lancet Public Health* **5**, e444-e451, doi:10.1016/S2468-2667(20)30146-8 (2020).
